# HTRA3 Is a Prognostic Biomarker and Associated With Immune Infiltrates in Gastric Cancer

**DOI:** 10.3389/fonc.2020.603480

**Published:** 2020-12-23

**Authors:** Ce Ji, Li-Sha Sun, Fei Xing, Nan Niu, Hong-Li Gao, Jing-Wei Dai, Nan Zhou, Ben-Chun Jiang

**Affiliations:** ^1^ Department of General Surgery, Shengjing Hospital of China Medical University, Shenyang, China; ^2^ Tumor Stem Cell and Transforming Medicine Laboratory, Shengjing Hospital of China Medical University, Benxi, China; ^3^ Department of Breast Surgery, Shengjing Hospital of China Medical University, Shenyang, China; ^4^ Department of Oncology, Shengjing Hospital of China Medical University, Shenyang, China; ^5^ Department of Neurosurgery, Shengjing Hospital of China Medical University, Shenyang, China

**Keywords:** HTRA3, bioinformatics, biomarker, gastric cancer, immune infiltration, prognosis

## Abstract

HtrA serine peptidase 3 (HTRA3) participates in multiple signal pathways and plays an important regulatory role in various malignancies; however, its role on prognosis and immune infiltrates in gastric cancer (GC) remains unclear. The study investigated HTRA3 expression in tumor tissues and its association with immune infiltrates, and determined its prognostic roles in GC patients. Patients with GC were collected from the cancer genome atlas (TCGA). We compared the expression of HTRA3 in GC and normal gastric mucosa tissues with Wilcoxon rank sum test. And logistic regression was used to evaluate the relationship between HTRA3 and clinicopathological characters. Gene ontology (GO) term analysis, Gene set enrichment analysis (GSEA), and single-sample Gene Set Enrichment Analysis (ssGSEA) was conducted to explain the enrichmental pathways and functions and quantify the extent of immune cells infiltration for HTRA3. Kaplan-Meier analysis and Cox regression were performed to evaluate the correlation between HTRA3 and survival rates. A nomogram, based on Cox multivariate analysis, was used to predict the impact of HTRA3 on prognosis. High HTRA3 expression was significantly correlated with tumor histological type, histological grade, clinical stage, T stage, and TP53 status (*P* < 0.05). HTRA3-high GC patients had a lower 10-year progression-free interval [PFI; hazard ratio (HR): 1.46; 95% confidence interval (CI): 1.02–2.08; *P* = 0.038], disease-specific survival (DSS; HR: 1.65; CI: 1.08–2.52; *P* = 0.021) and overall survival (OS; HR: 1.59; CI: 1.14–2.22; *P* = 0.006). Multivariate survival analysis showed that HTRA3 was an independent prognostic marker for PFI (HR: 1.456; CI: 1.021–2.078; *P* = 0.038), DSS (HR: 1.650; CI: 1.079–2.522; *P* = 0.021) and OS [hazard ratio (HR): 1.590; 95% confidence interval (CI):1.140–2.219; *P* = 0.006]. The C-indexes and calibration plots of the nomogram based on multivariate analysis indicated an effective predictive performance for GC patients. GSEA showed that High HTRA3 expression may activate NF-κB pathway, YAP1/WWTR1/TAZ pathway, and TGFβ pathway. There was a negative correlation between the HTRA3 expression and the abundances of adaptive immunocytes (T helper cell 17 cells) and a positive correlation with abundances of innate immunocytes (natural killer cells, macrophages etc.). HTRA3 plays a vital role in GC progression and prognosis and could be a moderate biomarker for prediction for survival after gastrectomy.

## Introduction

Gastric cancer (GC) is one of the most lethal carcinomas worldwide, and almost half of all GC cases are diagnosed in East Asia (1). Despite the developments in multimodal therapy strategies, such as surgery, chemotherapy, radiotherapy and immunotherapy, the five-year survival rates for the patients with stage II and stage IIIA GC were about 34 and 20%, respectively (2). GC is a heterogeneous malignancy (2, 3) and the existing microsatellite instability, HER2 mutation, and amplification cannot completely explain the different prognosis or therapeutic response of GC (4, 5). And the current biomarkers for the prognosis of GC are not suitable for clinical needs. Numerous studies currently on molecular targeted therapy and associated molecular pathways referred to the gastric tumorigenesis have illuminated the pathogenesis of GC and helped ameliorate the prognosis of patients with GC (6). For example, Trastuzumab can extend the survival time of the patients with HER2-positive GC (7). Therefore, there is an urgent necessity for the elucidation of the identification of novel biomarkers for carcinoma diagnosis and therapeutic targets in GC.

HtrA Serine Peptidase 3 (HTRA3), which was first reported in 2003, is a protein encoding gene located on chromosome 4p16.1 (8). HTRA3 protein (expressed in many cell types and organs) has two isomers, which both represent active serine proteases which cleave beta-casein/CSN2 as well as several extracellular matrix (ECM) proteoglycans (9). Previous studies indicated that HTRA3 inhibits signaling mediated by TGF-beta family proteins possibly indirectly by degradation of these ECM proteoglycans (10).

HTRA3 was first shown to be associated with cancer in a report demonstrating that it may act as a tumor suppressor, which is a pro-apoptotic protease that promotes drug-induced cytotoxic effects in lung cancer cells (11). Overexpressed HTRA3 inhibits the carcinogenic role of TGFβ1 and thus inhibits metastasis in the early stages of non-small cell lung cancer (10). HTRA3 expression was negatively correlated with lymph node metastasis in breast cancer, but not with positive or negative expression of ER and PR (12). Recent studies illuminated it represents as a tumor promoter. The expression of HTRA3 in the peritumor stroma of patients with stage II colorectal cancer is associated with high-grade tumor budding, which may be a new marker of poor prognosis (13). HTRA3 may be related to the acquisition of invasive phenotype of and may be a potential prognostic indicator of oral cancer (14). These findings suggest that HTRA3 has multifaceted functional roles in various malignancies. Moreover, the underlying functions and mechanisms of HTRA3 in tumor progression and tumor immunology are still unclear.

In this study, we aimed to systematically analyze the significance of HTRA3 in GC using RNA sequencing data retrieved from TCGA database, along with bioinformatics and statistical methods including differentially expressed genes (DEG) analysis, gene ontology (GO) term analysis, Kyoto Encyclopedia of Genes and Genomes (KEGG) pathway analysis, gene set enrichment analysis (GSEA), single-sample gene set enrichment analysis (ssGSEA), Kaplan-Meier survival analysis, and logistic & Cox regression analysis. Moreover, we further developed a nomogram to predict the patients’ prognosis.

## Material and Methods

### Data Source and Preprocessing

Gene expression data with clinical information from STAD projects (included 32 normal and 375 tumor tissues, Workflow Type: HTSeq-FPKM) were collected from TCGA. The exclusion criteria were normal STAD samples and an overall survival less than 30 days. Next, level 3 HTSeq-FPKM data were transformed into TPM (transcripts per million reads), and the TPM data of 375 GC patients were used for further analyses. Unavailable or unknown clinical features were regarded as missing values. The data are summarized in **Supplemental Table 1**.

### HTRA3 Differential Expression in GC Tissues in the TCGA Database

Boxplots and scatter plots, using disease state (tumor or normal) as the variable, were generated to calculate differential expression of HTRA3. The diagnostic performance of HTRA3 was estimated using receiver operating characteristic (ROC) curves. Statistical ranking for HTRA3 expression above or below the median value was defined as HTRA3-high or HTRA3-low, respectively.

### Experimental Verification of HTRA3 Differential Expression in GC Tissues and Cell Lines With Quantitative Polymerase Chain Reaction (qPCR) and Western Blotting

A total of 44 pairs of human GC samples and non−malignant gastric tissues which at least 5 cm away from the tumor samples were collected from patients who underwent a gastrectomy at the Department of Surgery, Shengjing Hospital, China Medical University between January 2019 and December 2019. All GC cases were pathologically confirmed. Five human GC cell lines (SGC7901, BGC823, MGC803, HGC27, and MKN45) and one immortalized normal gastric cell line (GES1) were obtained from the Institute of Biochemistry and Cell Biology, Chinese Academy of Sciences (Shanghai, P.R. China). Total RNA and proteins were obtained from these specimens.

The expression of HTRA3 mRNA was detected using qPCR with the following program: 95˚C for 30 s, 40 cycles of 95°C for 5 s, and 60°C for 30 s. The reaction mixture contained 10 μl SYBR Green (Takara, Dalian, China), 0.4 μl each primer, 2 μl cDNA, and 7.2 μl diethylpyrocarbonate (DEPC)−treated water. Real-time PCR was performed according to the protocol of SYBR Premix Ex TaqTMII kit. The primers used were as follows: Sense: 5’ −CTGAGACACCCGCTGTTTG− 3’ and antisense: 5’ −CCATTCTGTAGCTGCACCTT− 3’ for HTRA3; and sense: 5’ −TGACTTCAACAGCGACACCCA−3’ and antisense: 5’−CACCCTGTTGCTGTAGCCAAA−3’ for GADPH. Gene expression levels were calculated relative to the housekeeping gene GAPDH.

Each sample (60 μg) was electrophoresed in 10% polyacrylamide gel and transferred to a polyvinylidene difluoride membrane (Millipore, Bedford, MA, USA) using a BG-blotMiMi transfer machine (Baygene, Beijing, China). After blocking with 5% nonfat milk for 2 h at room temperature (about 20 to 25°C.), overnight incubation with primary antibodies for HTRA3 (1:500 dilution; Abcom) or GAPDH (1:2,000 dilution; Santa Cruz) was performed at 4°C. The next day, after incubation with secondary antibodies (1:5000; Santa Cruz) at 37°C for 2 h, and after washing, the immunoreactive protein bands were visualized using an electrochemiluminescence (ECL) detection kit (Thermol Biotech, Rockford, IL, USA). The ratio between the optical density of the protein and GAPDH was calculated as the relative content of the protein detected. Each experiment was repeated three times.

### Analysis of DEGs Between HTRA3-High and -Low Expression GC Groups

DEGs between HTRA3-high and HTRA3-low patients from TCGA datasets were identified by the unpaired Student’s t-test, within the DESeq2 (3.8) package (15). Genes with the adjusted *P* value <0.05 and the absolute FC larger than 1.5 were considered to be statistically significant. All the DEGs were presented in a heat map and volcano plots.

### Functional Enrichment and Analysis of Immune Cell Infiltration

In this study, Metascape (http://metasape.org) (16) was used as a tool to analyze the enrichment of HTRA3 related DEGs by process and pathway. The threshold conditions included: *P <*0.01, a minimum count of 3, and the enrichment factor >1.5 to obtain significant statistical differences.

GSEA starts with the HTRA3 differentially expressed matrix and analyzes the differences in signal pathways between the HTRA3-high and -low groups to predict the HTRA3-related phenotypes and signal pathways. A permutation test with 1,000 times was used to identify the significantly changed pathways. Adjusted P <0.01 and FDR <0.25 were identified as significant related genes. Statistical analysis and graphical plotting were conducted using R package clusterProfiler (3.8.0) (17). To construct the protein-protein interaction (PPI) network, the DEGs were input into STRING database (18). And PPI pairs with an interaction score >0.95 were chosen to build the PPI network.

The relative tumor infiltration levels of 24 immune cell types were quantified by ssGSEA to interrogate expression levels of genes in published signature gene lists (19). The signatures we used included a diverse set of adaptive and innate immune cell types and comprised 509 genes in total. To explore the correlation between HTRA3 and the infiltration levels of immune cells and the association of infiltration of immune cells with the different expression groups of HTRA3, Wilcoxon rank sum test, and Spearman correlation were adopted.

### Clinical Statistical Analysis on Prognosis, Model Construction, and Evaluation

All statistical analyses were performed in R package (V3.6.2). The relationship between clinical pathologic features and HTRA3 were analyzed with the Wilcoxon signed-rank sum test and logistic regression. Clinicopathological characteristics associated with the 10-year overall survival (OS), progression-free interval (PFI), and disease-specific survival (DSS) in TCGA patients using Cox regression and the Kaplan-Meier method. Multivariate Cox analysis was used to compare the influence of HTRA3 expression on survival along with other clinical characteristics (stage, myometrial invasion, lymph node status, distant metastasis status, histological grade and subtype). The cut-off value of HTRA3 expression was determined by its median value. P-values less than 0.05 were considered significant in all tests. The difference of 10-year OS, PFI, and DSS between HTRA3-high and -low group was calculated by the Kaplan-Meier method with a two-sided log-rank test.

Based on Cox regression models, the independent prognostic factors obtained from multivariate analysis were used to establish nomograms, individualizing the predicted survival probability for 1-, 3-, and 5-year. The RMS package (Version: 5.1-4; https://cran.r-project.org/web/packages/rms/index.html) was employed to generate nomograms that included significant clinical characteristics and calibration plots. The calibration curves were graphically assessed by mapping the nomogram-predicted probabilities against the observed occurrences, and the 45°line represented the best predictive values. A concordance index (C-index) was used to determine the discrimination of the nomogram, and it was calculated by a bootstrap approach with 1,000 resamples. The predictive accuracies of the nomogram and separate prognostic factors were compared using the C-index. All statistical tests were two tailed with a statistical significance level set at 0.05 in this study.

## Results

### Abnormally High Expression of HTRA3 in GC

**Figure 1:**
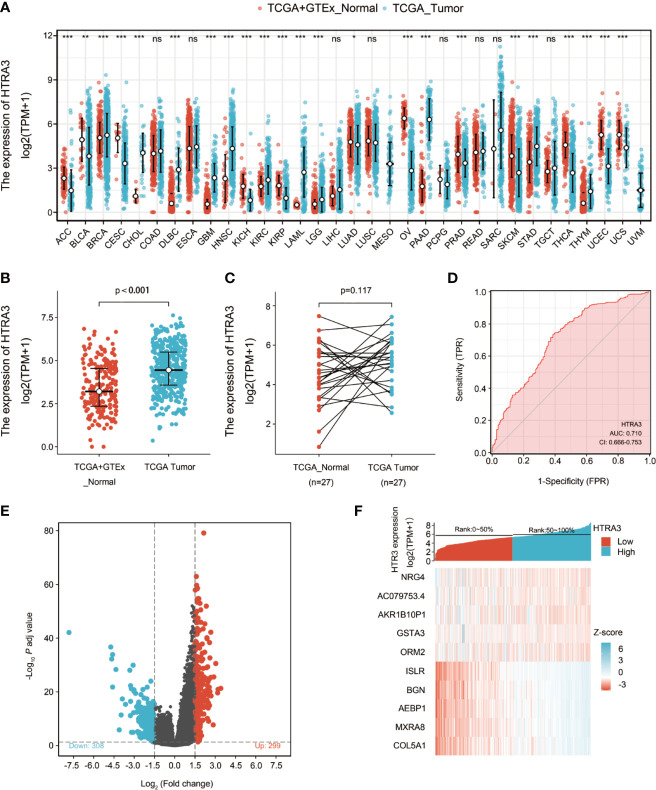
Differential expression levels of HTRA3 in different malignancies and HTRA3-related differentially expressed genes (DEGs). **(A)** Increased or decreased HTRA3 of different cancers compared with normal tissues in the TCGA and GTEx database. **(B, C)** Differential expression levels of HTRA3 in GC. **(D)** A ROC curve to test the value of HTRA3 to identify GC tissues was created. **(E, F)** Volcano plots of the DEGs and heat map showing the top 10 DEGs.

Firstly, the pan-cancer analyses were performed to compare the expression of HTRA3 in the tumor samples of GTEx combined with TCGA and the corresponding normal samples of TCGA by Wilcoxon rank sum test. HTRA3 was significantly expressed in adrenocortical carcinoma (ACC), bladder urothelial carcinoma (BLCA), breast infiltrating carcinoma (BRCA), cervical squamous cell carcinoma and adenocarcinoma (CESC), cholangiocarcinoma (CHOL), diffuse large B cell lymphoma (DLBCL), pleomorphic glioma (GBM), head and neck squamous cell carcinoma (HNSC), renal chromophobe cell carcinoma (KICH), renal clear cell carcinoma (KIRC), renal papillary cell carcinoma (KIRP), acute myeloid leukemia (LAML), brain low grade glioma (LGG), lung adenocarcinoma (LUAD), ovarian serous cystadenocarcinoma (OV), Pancreatic cancer (PAAD), prostate cancer (PRAD), skin melanoma (SKCM), gastric cancer (STAD), thyroid cancer (THCA), thymic cancer (THYM), endometrial cancer (UCEC), uterine sarcoma (UCS) (*P* < 0.05) (**Figure 1A**). Secondly, we compared the expression of HTRA3 in 32 paracancerous samples and 375 GC samples in TCGA STAD dataset. The expression of HTRA3 was significantly high in GC samples (*P* = 0.002) (**Figure 1B**; **Supplementary Table 1**). However, there was no significant difference in the expression of HTRA3 in 27 GC samples and matched paracancerous samples (p = 0.117) (**Figure 1C**), that may be due to the small number of paired samples in TCGA database. Therefore, we expanded the number of paired samples to verify mRNA and protein differential expression of HTRA3 in gastric cancer tissues and cell lines. We verified that HTRA3 is highly expressed in GC tissues by QPCR (*P* = 0.0035) and WB analysis (*P* = 0.0013) (**Supplementary Figure 1A**) on 44 paired GC samples and paracancerous samples. Among the GC cells, HTRA3 mRNA and protein expression was found at a comparatively higher level in MGC803, HGC27, and MKN45 cells and was lowest in SGC7901 (**Supplementary Figure 1B**).

ROC was used to analyze the distinguishing efficacy of HTRA3 between GC tissues and normal gastric mucosa tissue. The area under the curve (AUC) of HTRA3 is 0.710, suggesting that HTRA3 may be a potentially moderate identification molecule for GC tissues (**Figure 1D**).

### Identification of DEGs in GC

We compared 187 GC HTRA3-high samples with 188 HTRA3-low controls. A total of 607 DEGs, covering 299 upregulated GENEs and 308 downregulated GENEs, were identified to be statistically significant between the two cohorts (adjusted p-value < 0.05, |Log2-fold change| > 1.5) (**Figure 1E**; **Supplementary Table 2**). Then, DEGs in HTSeq-Counts were further analyzed by DESeq2 package. Relative expression values of the top 10 DEGs between the two cohorts were showed in **Figure 1F**.

### Functional Enrichment and Analyses of HTRA3 Related Genes in GC

**Figure 2:**
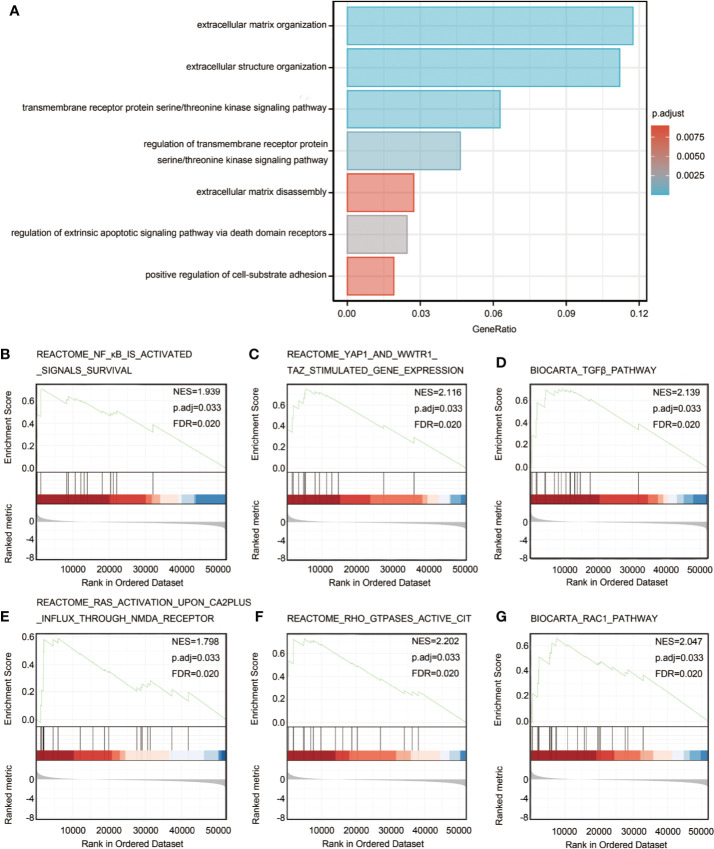
Significantly enriched GO annotations of HTRA3 related genes in GC. **(A)** Top 7 of biological process enrichment related to HTRA3 related genes with bar graph. **(B–G)** Enrichment plots from the gene set enrichment analysis (GSEA). Several pathways and biological processes were differentially enriched in HTRA3-related GC, including activated NF-κB signals survival, YAP1 and WWTR1-TAZ stimulated gene expression, TGFβ pathway, RAS activation upon Ca^2+^ influx through NMDA receptor, active CIT activated by RHO GTPases, and RAC1 signaling pathway. NES, normalized enrichment score; p.adj, adjusted *P* value; FDR, false discovery rate.

In order to predict the functional enrichment information of HTRA3 interactive genes, we used Metascape for GO enrichment analysis, which showed that HTRA3-related genes were involved in many biological processes (BPs), cellular compositions (CCs), and molecular functions (MFs), including extracellular matrix organization, extracellular structure organization, transmembrane receptor protein serine/threonine kinase signaling pathway. Moreover, regulation of transmembrane receptor protein serine/threonine kinase signaling pathway, extracellular matrix disassembly, regulation of extrinsic apoptotic signaling pathway *via* death domain receptors, and positive regulation of cell-substrate adhesion were also involved in the regulation of HTRA3 interactive genes (**Figure 2A**; **Supplementary Table 3**).

### Protein-Protein Interaction (PPI) Network Analysis

To obtain the interactions between the 607 DEGs in the GC group, a PPI network was constructed using the STRING database and the interaction threshold is set at 0.90. A total of 319 proteins and 1,425 edges were selected and 4 hub gene clusters were selected from PPI network with scores ≥6,600 (**Supplementary Figures 2A–E**). Additionally, top 10 hub genes included *IVL*, *TGM1*, *SPRR3*, *SPRR2A*, *SPRR2E*, *SPRR2D*, *LCE3E*, *SPRR2B*, *LCE3D*, and *SPRR2G*.

### GSEA Identifies HTRA3-Related Signaling Pathways

To identify HTRA3-related signaling pathways in GC, GSEA between HTRA3-high and -low expression data sets was conducted to reveal significant differences (adjusted *P* < 0.05, FDR q value < 0.25) in enrichment of MSigDB Collection (c2.cp.biocarta and hall. v6.1 symbols). The most significantly enriched signaling pathways based on their normalized enrichment score (HES) were selected. Moreover, the differentially enriched pathways in HTRA3 low expression phenotype include NF-κB signaling pathway, YAP1/WWTR1/TAZ signaling pathway, TGFβ pathway, RAS/CA2P/NMDA pathway, RHO/CIT pathway, and RAC1 signaling pathway (**Figures 2B–G**; **Supplementary Table 5**).

### The Correlation Between HTRA3 Expression and Immune Infiltration

**Figure 3:**
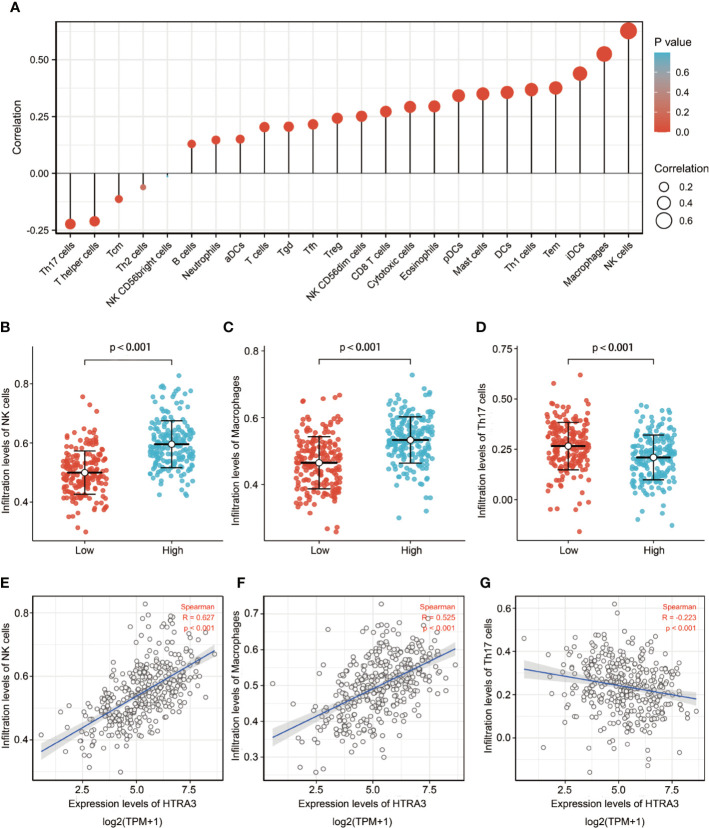
The expression level of HTRA3 was associated with the immune infiltration in the tumor microenvironment. **(A)** Correlation between the relative abundances of 24 immune cells and HTRA3 expression level. The size of dots shows the absolute value of Spearman R. **(B–G)** Scatter plots and correlation diagrams showing the difference of NK cells, Macrophages, and Th17 cells infiltration level between HTRA3-high and -low groups.

The correlation between the expression level (TPM) of HTRA3 and immune cell infiltration level quantified by ssGSEA was analyzed by spearman correlation. The expression of HTRA3 was negatively correlated with the abundance of acquired immunocytes [helper T17 (Th17) cells, T helper cells, T central memory cells, etc.], and positively correlated with the abundance of innate immunocytes [natural killer (NK) cells, tumor-associated macrophages (TAMs), immature dendritic cells, etc.] (**Figures 3A–G**, *P* < 0.001).

### Association With HTRA3 Expression and Clinicopathological Variables

**Figure 4:**
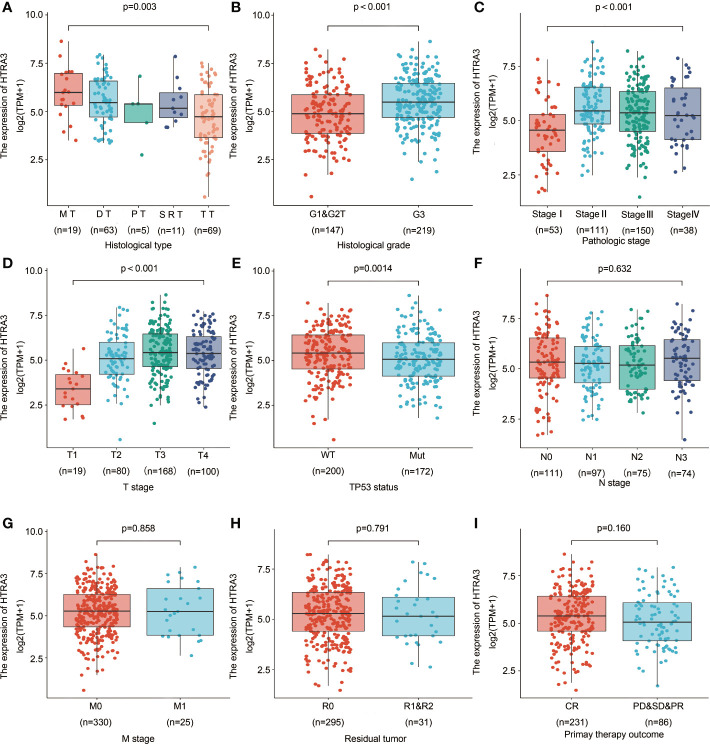
Association with HTRA3 expression and clinicopathological characteristics, including **(A)** histological type, **(B)** histological grade, **(C)** pathologic stage, **(D)** T stage **(E)** TP53 status, **(F)** N stage, **(G)** M stage, **(H)** residual tumor, and **(I)** primary therapy outcome in GC patients in TCGA cohort. TCGA, The Cancer Genome Atlas; GC, gastric cancer; MT, mucinous type; DT, diffuse type; PT, papillary type; SRT, signet ring type; TT, tubular type.

To clarify the role and significance of HTRA3 expression, a total of 375 GC samples with HTRA3 expression data with all patients’ characteristics were analyzed from TCGA. The cohort included 241 men and 134 women with an average age of 61.1 years (range 51–69 years). As shown in **Figures 4A–E** and **Table 1**, overexpressed HTRA3 was significantly correlated with tumor histological type (mucinous type vs. tubular type, *P* = 0.003), histological grade (grade 3 vs. grade 1 and 2, *P* < 0.001), clinical stage (stage IV vs. stage I, *P* < 0.001), T stage (T4 vs. T1, *P* < 0.001), and TP53 status (wild type vs. mutational type, *P* = 0.014). HTRA3 expression has no relation with other clinicopathological characteristics (**Figures 4F–I**). The univariate analysis with Logistic regression illuminated HTRA3 expression as a categorical dependent variable was associated with poor prognostic clinicopathological characteristics (**Table 2**). Increased HTTA3 expression in GC is positively associated with T stage (OR = 2.39 for T3 and T4 vs. T1 and T2), histological grade (OR = 2.14 for G3 vs. G1 and G2); meanwhile negatively associated with TP53 status (OR = 0.59 for mutation vs. wild type) significantly (all *P* < 0.05). These results suggested that GCs with high HTRA3 expression were prone to progress to a more advanced stage and less susceptible to TP53 mutations than those with low HTRA3 expression.

**Table 1:** The association between HTRA3 expression and clinicopathological variables. Abbreviations: ^a^ statistically significant ^b^ Exact χ^2^ test.

Characters	level	Low expression of HTRA3	High expression of HTRA3	p	test
N		188	187		
T stage (%)	T1	18(9.8%)	1(0.5%)	<0.001	exact
	T2	47(25.5%)	33(18.0%)		
	T3	72(39.1%)	96(52.5%)		
	T4	47(25.5%)	53(29.0%)		
N stage (%)	N0	53(29.8%)	58(32.4%)	0.589	exact
	N1	52(29.2%)	45(25.1%)		
	N2	40(22.5%)	35(19.6%)		
	N3	33(18.5%)	41(22.9%)		
M stage (%)	M0	167(92.8%)	163(93.1%)	1.000	exact
	M1	13(7.2%)	12(6.9%)		
Pathologic stage (%)	Stage I	38(21.7%)	15(8.5%)	0.002	exact
	Stage II	46(26.3%)	65(36.7%)		
	Stage III	70(40.0%)	80(45.2%)		
	Stage IV	21(12.0%)	17(9.6%)		
Tumor status (%)	Tumor free	108(64.7%)	106(62.4%)	0.734	exact
	With tumor	59(35.3%)	64(37.6%)		
Primary therapy outcome (%)	CR	106(68.4%)	125(77.2%)	0.342	exact
	PD	38(24.5%)	27(16.7%)		
	PR	2(1.3%)	2(1.2%)		
	SD	9(5.8%)	8(4.9%)		
Gender (%)	Female	65(34.6%)	69(36.9%)	0.718	
	Male	123(65.4%)	118(63.1%)		
Race (%)	Asian	41(26.8%)	33(19.4%)	<0.001	
	Black or African American	11(7.2%)	0(0.0%)		
	White	101(66.0%)	137(80.6%)		
Age (%)	<=65	78(42.2%)	86(46.2%)	0.493	
	>65	107(57.8%)	100(53.8%)		
Histological type (%)	Diffuse Type	28(15.0%)	35(18.7%)	0.031	exact
	Mucinous Type	5(2.7%)	14(7.5%)		
	Not Otherwise Specified	101(54.0%)	106(56.7%)		
	Papillary Type	2(1.1%)	3(1.6%)		
	Signet Ring Type	6(3.2%)	5(2.7%)		
	Tubular Type	45(24.1%)	24(12.8%)		
Residual tumor (%)	R0	147(89.6%)	151(91.5%)	0.811	exact
	R1	8(4.9%)	7(4.2%)		
	R2	9(5.5%)	7(4.2%)		
Histologic grade (%)	G1	5(2.7%)	5(2.7%)	0.001	exact
	G2	85(46.4%)	52(28.4%)		
	G3	93(50.8%)	126(68.9%)		
Anatomic neoplasm subdivision (%)	Antrum/Distal	65(36.1%)	73(40.3%)	0.043	exact
	Cardia/Proximal	25(13.9%)	23(12.7%)		
	Fundus/Body	59(32.8%)	71(39.2%)		
	Gastroesophageal Junction	27(15.0%)	14(7.7%)		
	Other	4(2.2%)	0(0.0%)		
Reflux history (%)	No	90(77.6%)	85(86.7%)	0.121	
	Yes	26(22.4%)	13(13.3%)		
Antireflux treatment (%)	No	82(82.0%)	60(75.9%)	0.420	
	Yes	18(18.0%)	19(24.1%)		
Barretts esophagus (%)	No	116(92.1%)	77(93.9%)	0.821	
	Yes	10(7.9%)	5(6.1%)		
TP53 status (%)	Mut	99(52.7%)	73(39.7%)	0.013	exact
	WT	89(47.3%)	111(60.3%)		
PIK3CA status (%)	Mut	25(13.3%)	34(18.5%)	0.202	exact
	WT	163(86.7%)	150(81.5%)		
Age [median (IQR)]		68.00[58.00,74.00]	67.00[58.00,72.00]	0.367	nonnorm

**Table 2:** HTRA3 expression association with clinical pathological characteristics (logistic regression).

Characteristics	Odds Ratio in HTRA3 expression	Odds Ratio (OR)	P value
T stage (T3&T4 vs. T1&T2)	367	2.39(1.49–3.90)	<0.001
N stage (N1&N2&N3 vs. N0)	357	0.88(0.56–1.39)	0.592
M stage (M1 vs. M0)	355	0.95(0.41–2.15)	0.893
Pathologic stage (Stage III & Stage IV vs. Stage I & Stage II)	352	1.12(0.74–1.70)	0.598
Histological type (Diffuse Type vs. Tubular Type)	132	2.34(1.17–4.78)	0.017
Histologic grade (G3 vs. G1&G2)	366	2.14(1.40–3.29)	<0.001
Primary therapy outcome (CR vs. PD&SD&PR)	317	1.56(0.95–2.58)	0.080
Tumor status (With tumor vs. Tumor free)	337	1.11(0.71–1.72)	0.659
Residual tumor (R1&R2 vs. R0)	329	0.80(0.38–1.68)	0.560
TP53 status (Mut vs. WT)	372	0.59(0.39–0.89)	0.012
PIK3CA status (Mut vs. WT)	372	1.48(0.85–2.61)	0.173

### High HTRA3 Expression Was Closely Associated With Poor Prognosis of Patients With GC

**Figure 5:**
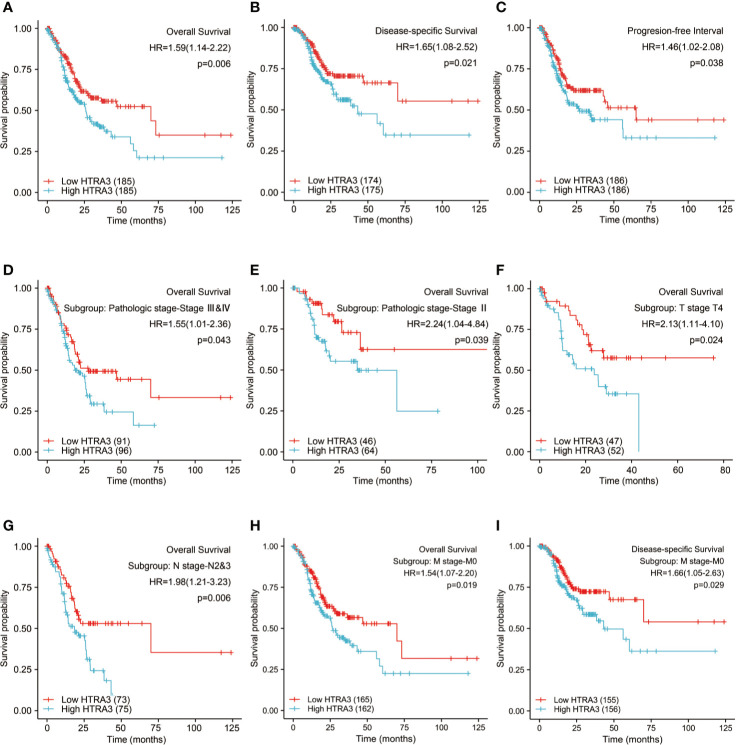
Kaplan-Meier survival curves comparing the high and low expression of HTRA3 in GC. **(A–C)** Survival curves of OS, DSS, and PFI between HTRA3-high and -low patients with GC. **(D–H)** OS survival curves of stage III & IV, stage III, T4, N2&3, and M0 subgroups between HTRA3-high and -low patients with GC. **(I)** DSS survival curves of M0 subgroup between HTRA3-high and -low patients with GC. GC, gastric cancer; OS, overall survival; DSS, disease specific survival; PFI, progression free interval.

The 10-year OS rates were significantly higher among patients with low HTRA3 expression than those with high HTRA3 expression (37.6 vs. 24.7%; *P* = 0.006; **Figure 5A**). Similarly, the 10-year PFI rates and DSS in the HTRA3-low group were significantly higher than those in the HTRA3-high group (46.9 vs. 36.7%; *P* = 0.038; 57.3 vs. 38.2%; *P* = 0.021; **Figures 5B, C**).

Next, we conducted subgroup survival analyses of OS, DSS, and PFI, which showed that the prognosis of patients with HTRA3-high was poor in stage III–IV, stage III, T4, N2&N3, and M0 subgroups of OS and M0 subgroup of DSS (**Figures 5D–I**). Furthermore, it should be noted that the GC patients with HTRA3-high in M0 subgroup had worse OS and DSS (33.2 vs. 22.7%; *P* = 0.019; 54.3 vs. 37.2%; *P* = 0.029), indicating HTRA3 had a greater prognostic role in GC patients without distant metastasis. However, there was no significant difference in survival among each subgroup of PFI.

A univariate logistic regression indicated that higher HTRA3 expression was associated with a short OS [hazard ratio (HR): 1.590; 95% confidence interval (CI):1.140–2.219; *P* = 0.006] and poor PFI (HR: 1.456; CI: 1.021–2.078; *P* = 0.038) as well as DSS (HR: 1.650; CI: 1.079–2.522; *P* = 0.021) (**Table 3** and **Supplemental Tables 6**, **7**). To further seek factors associated with survival, a multivariate Cox regression analysis was performed with pathologic stage, T stage, N stage, M stage, Histological grade, age, primary therapy outcome, and residual tumor status. High HTRA3 expression was still an independent factor associated with poor OS (HR: 2.315; CI: 1.447–3.703; *P* < 0.001) (**Table 3**). However, HTRA3 expression levels showed no association with poor DSS (HR: 1.405; CI: 0.824–2.395; *P* = 0.211) (**Supplemental Table 6**) and short PFI (HR: 1.116; CI: 0.656–1.898; *P* = 0.686) (**Supplemental Table 7**) in patients with GC.

**Table 3:** Univariate regression and multivariate survival method (Overall Survival) of prognostic covariates in patients with gastric cancer.

Characteristics	Total(N)	HR (95% CI) Univariate analysis	P value Univariate analysis	HR (95% CI) Multivariate analysis	P value Multivariate analysis
T stage (T3&T4 vs. T1&T2)	362	1.719(1.131–2.612)	0.011	1.103(0.582–2.092)	0.763
N stage (N1&N2&N3 vs. N0)	352	1.925(1.264–2.931)	0.002	2.394(1.051–5.452)	0.038
M stage (M1 vs. M0)	352	2.254(1.295–3.924)	0.004	0.677(0.263–1.744)	0.419
Pathologic stage (Stage III & Stage IV vs. Stage I & Stage II)	347	1.947(1.358–2.793)	<0.001	0.852(0.423–1.717)	0.655
Histologic grade (G3 vs. G1&G2)	361	1.353(0.957–1.914)	0.087	1.273(0.783–2.071)	0.330
Histological type (Diffuse Type vs. Tubular Type)	132	1.077(0.620–1.872)	0.793		
Primary therapy outcome (CR vs. PD&SD&PR)	313	0.237(0.163–0.344)	<0.001	0.426(0.241–0.755)	0.003
Residual tumor (R1&R2 vs. R0)	325	3.445(2.160–5.494)	<0.001	1.503(0.760–2.971)	0.241
Age (>65 vs. <=65)	367	1.620(1.154–2.276)	0.005	1.921(1.215–3.036)	0.005
Race (Asian & Black or African American vs. White)	320	0.801(0.515–1.247)	0.326		
Gender (Male vs. Female)	370	1.267(0.891–1.804)	0.188		
Anatomic neoplasm subdivision (Fundus/Body vs. Antrum/Distal)	267	0.965(0.651–1.430)	0.858		
Reflux history (Yes vs. No)	213	0.582(0.291–1.162)	0.125		
Antireflux treatment (Yes vs. No)	179	0.756(0.422–1.353)	0.346		
Barretts esophagus (Yes vs. No)	207	0.892(0.326–2.441)	0.824		
TP53 status (Mut vs. WT)	367	0.865(0.621–1.205)	0.392		
PIK3CA status (Mut vs. WT)	367	0.623(0.370–1.048)	0.075	0.468(0.248–0.881)	0.019
Tumor status (With tumor vs. Tumor free)	333	5.420(3.640–8.071)	<0.001	3.526(1.981–6.276)	<0.001
HTRA3 (High vs. Low)	370	1.590(1.140–2.219)	0.006	2.315(1.447–3.703)	<0.001

Based on the independent adverse prognostic factors chosen by multivariate Cox analysis, we studied the effect of HTRA3 expression on prognosis (OS, PFI, and DSS) in different subgroups. The HTRA3-high GC patients had shorter OS time in the T4 subgroup (HR: 2.130; CI: 1.106–4.104; *P* = 0.024), N2&3 subgroup (HR: 1.979; CI: 1.214–3.227; *P* = 0.006), M0 subgroup (HR: 1.536; CI: 1.075–2.196; *P* = 0.019) and pathologic stage III subgroup (HR: 2.244; CI: 1.040–4.838; *P* = 0.039) and stage III & IV subgroup (HR: 1.546; CI: 1.013–2.357; *P* = 0.043) (**Table 4**). Similarly, the DSS prognostic analyses showed that the HTRA3-high GC patients had a shorter survival time in M0 subgroup (HR: 1.665; CI: 1.055–2.627; *P* = 0.029) (**Supplemental Table 8**). However, the PFI prognostic analyses did not find that the difference in HTRA3 expression had a significant effect on the prognosis in any subgroup (**Supplemental Table 9**).

**Table 4:** The prognostic value of HTRA3 (Overall Survival) in various gastric cancer subgroups.

Characteristics	N (%)	HR (95% CI)	P value
T stage			
T1&T2	96 (27)	1.406(0.667–2.963)	0.370
T3	167 (46)	1.254(0.780–2.016)	0.349
T4	99 (27)	2.130(1.106–4.104)	0.024
N stage			
N0	107 (30)	1.460(0.666–3.198)	0.344
N1	97 (28)	1.362(0.736–2.521)	0.325
N2&N3	148 (42)	1.979(1.214–3.227)	0.006
M stage			
M0	327 (93)	1.536(1.075–2.196)	0.019
M1	25 (7)	1.551(0.527–4.564)	0.425
Pathologic stage			
Stage I	50 (14)	0.977(0.281–3.394)	0.971
Stage II	110 (32)	2.244(1.040–4.838)	0.039
Stage III & Stage IV	187 (54)	1.546(1.013–2.357)	0.043

### Construction and Validation of a Nomogram Based on the HTRA3

**Figure 6:**
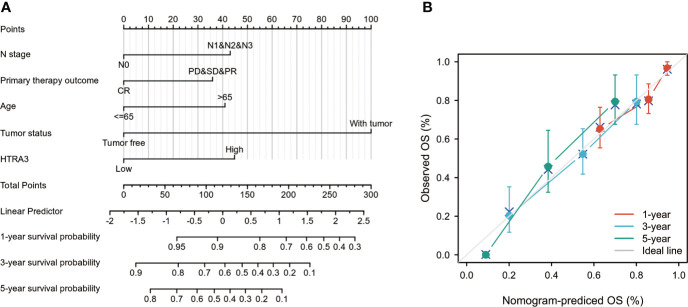
A quantitative method to predict GC patients’ probability of 1-, 3-, and 5-year OS. **(A)** A nomogram for predicting the probability of 1-, 3-, and 5- year OS for GC patients. **(B)** Calibration plots of the nomogram for predicting the probability of OS at 1, 3, and 5 years. GC, gastric cancer; OS, overall survival.

To provide a quantitative approach predicting the prognosis of GC patients, HTRA3 and independent clinical risk factors were used to construct a nomogram (**Figure 6A**). In the nomogram based on multivariate Cox analysis, a point scale was used to assign points to these variables. The sum of points assigned to each variable was readjusted to a range from 1 to 100. The points of the variables were accumulated and recorded as the total scores. The probability of survival in GC patients at 1, 3, and 5 years was determined by drawing a vertical line directly down from the total point axis to the outcome axis. For instance, a GC patient with high HTRA3 risk (45 points), lymph node metastasis (42.5 points), and poor therapy outcome (37 points) received a total point score of 124.5. The probabilities of 1-, 3-, 5-year survival were about 87.5, 57, and 38% (**Figure 6A**).

We also analyzed the prediction efficiency of the nomogram, and the result illuminated that the C-index of the model was 0.743(CI: 0.720–0.766), which suggested that the prediction efficiency of this model is moderately accurate. The bias-corrected line in the calibration plot was utilized to be close to the ideal curve (the 45-degree line), which showed a fine agreement between the prediction and the observation (**Figure 6B**). Moreover, the nomogram performance of HTRA3 (C-index: 0.743) was better than the performance of T stage (C-index: 0.698), M stage (C-index: 0.677), pathological stage (C-index: 0.722). These results suggested that the nomogram was a better model for predicting short- or long-term survival in patients with GC than individual prognostic factors.

## Discussion

To our knowledge, the expression of HTRA3 and its potential prognostic impact on GC has not been explored. Hence, the potential role of HTRA3 in GC was the focus of the present study. In the present study, bioinformatics analysis using high-throughput RNA-sequence data from TCGA database demonstrated that there were evident individual variation and heterogeneity in these RNA transcripts, and HTRA3 might be a potential moderate marker for GC tissues. Increased expression of HTRA3 in GC was associated with fewer TP53 mutations, advanced clinical pathologic characteristics, shorter survival time, and poor prognosis.

Human HTRA3 is a trimer multitasking serine protease related to cell function and pathogenicity. It may serve as a potential therapeutic target (9). HTRA3 dissolves XIAP and BAX, therefore promoting apoptosis. Additionally, it is associated with epithelial-mesenchymal transformation (EMT) (11). HTRA3 may inhibit the signal transduction of TGF-β family members and prohibit cell migration through MEK/ERK pathway (20). HTRA3 may promote the instability of actin and vimentin cytoskeleton and affect the dynamics of cytoskeleton (21).

In some malignancies, HTRA3 has anti-tumor effect. Previous studies have elucidated the low expression of HTRA3 in endometrial carcinoma and ovarian cancer had anti-tumor effect (22, 23). The ectopic expression of HTRA3 leaded to the decrease of cell proliferation and the increase of the expression of apoptotic protein Bax, suggesting that HTRA3 has anti-tumor effect on pancreatic cancer cells (24). The expression of HTRA3 in lung cancer tissue was significantly down-regulated. HTRA3 promoted drug-induced apoptosis of lung cancer cells (11). The expression of HTRA3 was negatively correlated with lymph node metastasis in breast cancer, but not with positive or negative expression of ER and PR (12). In non-small cell lung cancer cell lines, exogenous transforming growth factor β-1 significantly reduced HTRA3 expression during EMT (10).

However, HTRA3 can promote tumor progression in other malignancies. recent studies have also shown that HTRA3 was highly expressed in thyroid carcinoma and related to the occurrence of thyroid cancer (25). HTRA3 was related to the acquisition of invasive phenotype of oral squamous cell carcinoma and may be a potential prognostic indicator of oral cancer (14). HTRA3 is highly expressed in the stroma of the invasive front of colorectal cancer. The high expression of HTRA3 in tumor core was significantly correlated with the decrease of 5-year overall survival rate. In addition, the expression of HTRA3 in the peritumor stroma of patients with stage II colorectal cancer is related to high grade tumorous budding, which may be a new marker of poor prognosis (13).

To further investigate the functions of HTRA3 in GC, we performed GO, GESA, and ssGSEA analyses using TCGA data. The results revealed that NF-κB signaling pathway, YAP1/WWTR1/TAZ pathway, TGFβ signaling pathway, RAS/CA2P/NMDA pathway, RHO/CIT pathway, and RAC1 signaling pathway were differentially enriched in the HTRA3-high phenotype and fewer abundance of adaptive immune cells were also observed. These data suggested that HTRA3 might serve as a potential prognostic marker and therapeutic target in GC.

HTRA3 might promote the instability of cytoskeleton, thereby regulating the EMT process of various tumor cells. And HTRA3 is a potential therapeutic target involved in various cancers (21). Researchers revealed that the modulation of HTRA3 in tumorigenesis might be dual, either inhibitory or promoting, depending on the specific tissues, stages of cancer progression etc. On one hand, HTRA3 expression is low in endometrial cancer, ovarian cancer, and lung cancer (23, 26). HTRA3 promotes drug-induced apoptosis through XIAP cleavage in lung cancer cells (11). In non-small cell lung cancer (NSCLS) cell lines, overexpression of HTRA3 inhibits the carcinogenesis of TGF-β1, thus inhibiting tumor metastasis in the early stage of cancer. The role of HTRA3 is weakened, and transforming growth factor β1 effectively promoted EMT without HTRA3 brakes in the later stage of cancer (10). The ectopic expression of HTRA3 in pancreatic cancer leads to the decrease of cell proliferation and the increase of the expression of apoptotic protein Bax, suggesting that HTRA3 has anti-tumor effect on pancreatic cancer cells (24). On the other hand, HTRA3 is highly expressed in oral squamous cell carcinoma, thyroid cancer and colorectal cancer, and the acquisition of invasive phenotype in oral squamous cell carcinoma and colorectal tumor is closely related to poor prognosis (13, 14, 25). Here, we found that HTRA3 is highly expressed in GC. The overexpression of HTRA3 was significantly correlated with histological type, histological grade, clinical stage, T stage, and TP53 status of gastric cancer. HTRA3-high patients with GC had worse histological types (diffuse type), lower tumor differentiation, later clinicopathological stages (especially greater primary tumor growth), and were less prone to have TP53 mutations, highlighting the potential role of HTRA3 in the development of GC.

Given the limited data on HTRA3 function, we performed functional annotation based on GO and GESA. We demonstrated that HTRA3-high phenotype was associated with NF-κB signaling pathway, YAP1/WWTR1/TAZ signaling pathway, TGFβ pathway, RAS/CA2P/NMDA pathway, RHO/CIT pathway, and RAC1 signaling pathway. Recent studies have elucidated that Enhanced activity of NF-κB signaling pathway can promote proliferation, metastasis, and angiogenesis of GC cells (27). YAP1/TAZ activity controls GC stem cells tumorigenic properties (28), indicating that HTRA3 may promote GC cell growth, metastasis, and poor survival *via* the NF-κB and YAP1/WWTR1/TAZ pathway. Additionally, the PPI network of HTRA3-associated genes was constructed in this study, and these genes were involved in various signaling pathways and biological processes. In future studies, we will further determine the correlation between HTRA3-associated genes and the prognosis of GC.

Stromal cells in tumor microenvironment can change the carcinogenic characteristics of tumor cells. Among them, tumor-infiltrating lymphocytes (TILs) play an important role in the occurrence and development of tumors (29, 30). TILs establish a complex intercellular interaction network, which helps improve and maintain the immunosuppressive microenvironment, promote immune escape, and thus promote tumor progression (31). Our results demonstrated there were more NK cells, TAMs, and fewer Th17 cells in the HTRA3-high GC group than those in the HTRA3-low GC group, suggesting the improvement of innate immunity was accompanied by the decrease of adaptive immunity. Furthermore, infiltrating NK cells and TAMs in the tumor microenvironment have strong immunosuppressive activity, which reduces the secretion of IFN-γ and induces T cell dysfunction (32, 33). Additionally, the subgroup with more Th17 cell infiltration in breast and ovarian cancer is less likely to have lymph node metastasis and more likely to have a better prognosis, which suggests that Th17 cells have anti-tumor effect (34–36). Therefore, our data elucidated the immunosuppression induced by fewer Th17 cells in the primary tumor microenvironment might result in a lower 10-year survival rate in HTRA3-high patients with GC.

High level of HTRA3 expression was correlated with poor prognosis of GC in stage III – IV, T4, N2-3, and M0 subgroups, with the highest HR for poor OS, DSS, and PFI when HTRA3 was highly expressed in GC. We found that the expression of HTRA3 remained a powerful predictor of prognosis within these subsets, suggesting that HTRA3 was independent of these important clinicopathological parameters. Subsequently, a nomogram with comprehensive evaluation combining HTRA3 with other important clinical patterns (HTRA3 status, N stage, primary therapy outcome, age status, and tumor status) was performed. Based on the calibration plot, there was a favorable consistency between the actual and predicted values for 1-, 3-, 5-year OS. Our model was constructed based on the complementary perspective for respective tumors and provided a personalized score for individual patients. Consequently, our nomogram could be a valuable new prognostic method for clinicians in the future.

Although these results improved our understanding of the relationship between HTRA3 and GC, there were some limitations. First, to clarify the specific role of HTRA3 in the development of GC comprehensively, several clinical factors and parameters should be considered, such as the details on treatments received by patients involved. However, this information was lacked or inconsistent in public databases because the experiments were performed in different centers. Second, the number of healthy subjects used as controls was considerably different from that of patients with cancer in the current study, hence additional studies were required to maintain a balance of sample size. Third, although multi-center study in public databases intends to complement the drawbacks of single center study, retrospective studies have their limitations, especially non-uniform intervening measures, and lacking of some information. Therefore, a prospective study should be performed in the future to avoid analysis bias arising due to the retrospective nature of the current study. Lastly, since the current study was performed based on RNA sequencing from TCGA database only, it is necessary to further study the direct mechanism of HTRA3 in GC.

In this study, we firstly reported that the high expression of HTRA3 was significantly associated with the progression, poor survival, and immune infiltration of GC, which might promote tumorigenesis through abnormal inflammation and immune response. HTRA3 has the potential to predict treatment outcomes and may become a new biomarker of GC. The mechanism of HTRA3 promoting the progression and metastasis of GC will be verified in further studies. This study provided a new and promising insight for further elucidating the clinicopathological significance and molecular pathogenesis of GC.

## Data Availability Statement

The original contributions presented in the study are included in the article/**Supplementary Material**. Further inquiries can be directed to the corresponding author.

## Ethics Statement

Ethical review and approval were not required for the study on human participants in accordance with the local legislation and institutional requirements. The patients/participants provided their written informed consent to participate in this study.

## Author Contributions

B-CJ contributed to concept and design of this article. CJ participated in manuscript writing. L-SS, FX, and NN participated in data collection and data analysis. H-LG, J-WD, and NZ analyzed and interpreted data. All authors contributed to the article and approved the submitted version.

## Funding

This work was supported by the Medical and Health Science Technology Planning Projects of Liaoning Province of China (20170540986 and 2019-ZD-0739), the Science and Technology Planning Project of Shenyang City (18-014-4-22) and 50 Talent Project of Shengjing Hospital of China Medical University.

## Conflict of Interest

The authors declare that the research was conducted in the absence of any commercial or financial relationships that could be construed as a potential conflict of interest.

## Supplementary Material

Supplemental Figure 1 Experimental verification of differential mRNA and protein expression of HTRA3 in GC samples and cell lines. **(A)** HTRA3 mRNA and protein expression in 44 paired adjacent noncancerous tissues and GC tissues. **(B)** HTRA3 mRNA and protein expression in GC cell lines (SGC7901, MKN45, MGC803, BGC823, and HGC27) and a gastric epithelial cell line (GES1). GAPDH was used as a loading control. Results are representative of three different experiments and data are expressed as mean ± standard deviation. HTRA3, HtrA serine peptidase 3; GC, gastric cancer.

Supplemental Figure 2 The protein to protein interactions network constructed by protein to protein interactions pairs based on the STRING database.

Supplemental Table 1 Clinical characteristics of gastric cancer patients based on TCGA.

Supplemental Table 2 50 items of HTRA3-related differential expressed Genes.

Supplemental Table 3 50 items of Gene Ontology enrichment analysis.

Supplemental Table 4 Gene sets enriched in phenotype high. NES, normalized enrichment score; p. adjusted, adjusted P-value.

Supplementary Table 5 50 items of protein to protein interactions of HTRA3.

Supplementary Table 6 Univariate regression and multivariate survival method (Disease Specific Survival) of prognostic covariates in patients with gastric cancer.

Supplementary Table 7 Univariate regression and multivariate survival method (Progression Free Interval) of prognostic covariates in patients with gastric cancer.

Supplementary Table 8 The prognostic value of HTRA3 (Disease Specific Survival) in various gastric cancer subgroups.

Supplementary Table 9 The prognostic value of HTRA3 (Progression Free Interval) in various gastric cancer subgroups.
