# Supplementary figures and images for: HTRA3 Is a Prognostic Biomarker and Associated With Immune Infiltrates in Gastric Cancer
Source: Front Oncol. 2020 Dec 23;10:603480. doi: 10.3389/fonc.2020.603480 (PMC7786138; doi:10.3389/fonc.2020.603480)

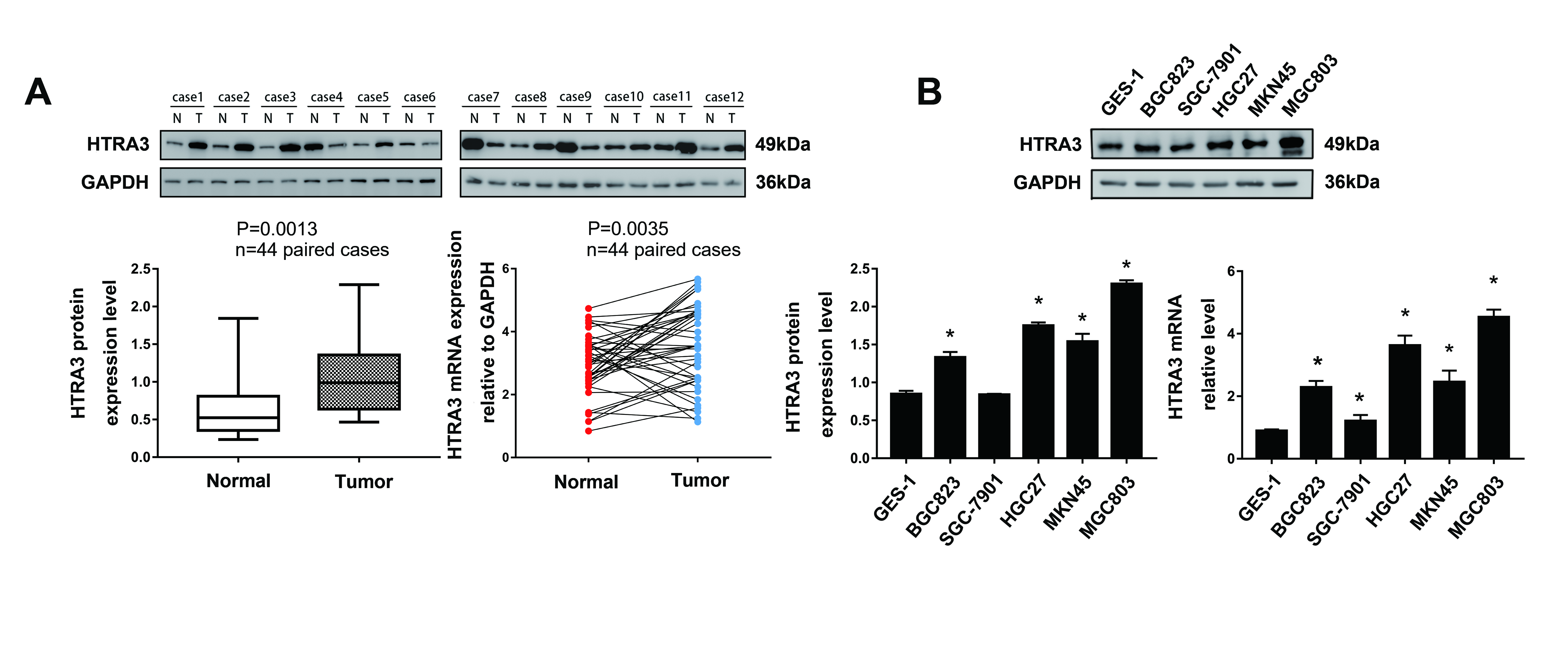

Supplement: Supplemental Figure 1 — Experimental verification of differential mRNA and protein expression of HTRA3 in GC samples and cell lines. (A) HTRA3 mRNA and protein expression in 44 paired adjacent noncancerous tissues and GC tissues. (B) HTRA3 mRNA and protein expression in GC cell lines (SGC7901, MKN45, MGC803, BGC823, and HGC27) and a gastric epithelial cell line (GES1). GAPDH was used as a loading control. Results are representative of three different experiments and data are expressed as mean ± standard deviation. HTRA3, HtrA serine peptidase 3; GC, gastric cancer. [file Image_1.jpeg]

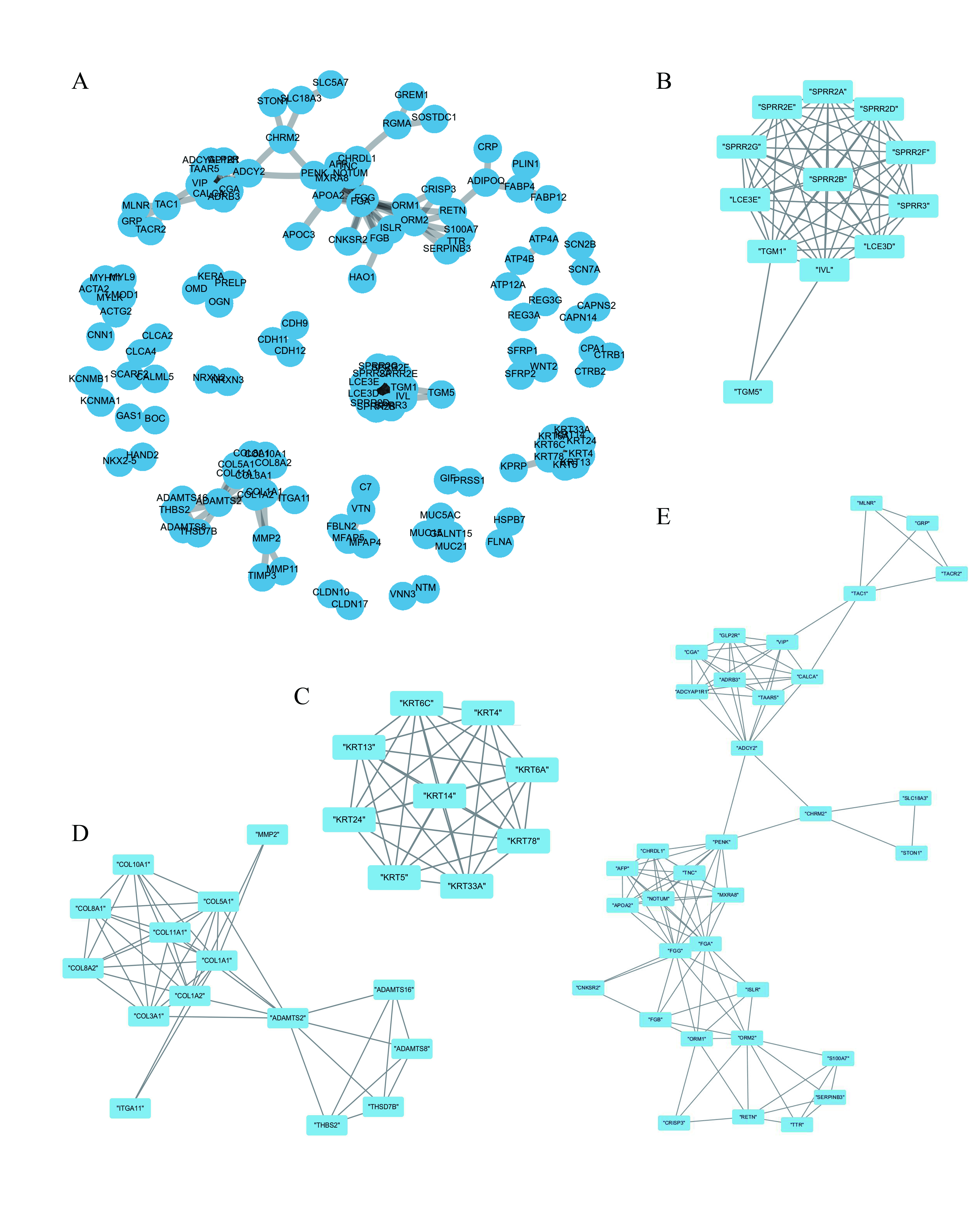

Supplement: Supplemental Figure 2 — The protein to protein interactions network constructed by protein to protein interactions pairs based on the STRING database. [file Image_2.jpeg]
