# Supplementary material for: HTRA3 Is a Prognostic Biomarker and Associated With Immune Infiltrates in Gastric Cancer
Source: Front Oncol. 2020 Dec 23;10:603480. doi: 10.3389/fonc.2020.603480 (PMC7786138; doi:10.3389/fonc.2020.603480)
Supplement: Supplemental Table 1 — Clinical characteristics of gastric cancer patients based on TCGA. [file DataSheet_1.zip › Supplemental Table 2ú║50 items of HTRA3-related differential expressed Genes..docx]

Supplemental Table 1：50 items of differential expressed Genes of HTRA3

| gene_id | baseMean | log2FoldChange | lfcSE | stat | pvalue | padj | gene_name | gene_biotype | cor_pvalue | correlation |
| --- | --- | --- | --- | --- | --- | --- | --- | --- | --- | --- |
| ENSG00000198804 | 1705067.72 | -0.327352041 | 0.07609405 | -4.30194024 | 1.693090e-05 | 1.624794e-04 | MT-CO1 | protein_coding | 1.539622e-02 | -0.125103197 |
| ENSG00000198886 | 1144371.35 | -0.382777531 | 0.07410732 | -5.16517824 | 2.402099e-07 | 4.056263e-06 | MT-ND4 | protein_coding | 5.155318e-08 | -0.277769940 |
| ENSG00000210082 | 974077.28 | -0.400835139 | 0.09583723 | -4.18245741 | 2.883750e-05 | 2.539219e-04 | MT-RNR2 | Mt_rRNA | 2.375097e-03 | -0.156634429 |
| ENSG00000198938 | 690481.27 | -0.403523700 | 0.08913363 | -4.52717653 | 5.977700e-06 | 6.724181e-05 | MT-CO3 | protein_coding | 6.008375e-04 | -0.176631926 |
| ENSG00000198727 | 634725.07 | -0.381869791 | 0.08527017 | -4.47835157 | 7.522164e-06 | 8.175639e-05 | MT-CYB | protein_coding | 3.617339e-06 | -0.237273638 |
| ENSG00000198712 | 564528.23 | -0.399098259 | 0.08562979 | -4.66074090 | 3.150732e-06 | 3.891490e-05 | MT-CO2 | protein_coding | 6.431033e-08 | -0.275817272 |
| ENSG00000198763 | 461951.17 | -0.374892566 | 0.07844616 | -4.77897895 | 1.761876e-06 | 2.342999e-05 | MT-ND2 | protein_coding | 3.309285e-05 | -0.213089771 |
| ENSG00000075624 | 447843.44 | 0.243986477 | 0.05622630 | 4.33936548 | 1.428947e-05 | 1.410338e-04 | ACTB | protein_coding | 0.000000e+00 | 0.413299807 |
| ENSG00000198888 | 395896.99 | -0.481642182 | 0.08387434 | -5.74242591 | 9.332972e-09 | 2.207871e-07 | MT-ND1 | protein_coding | 1.766504e-07 | -0.266699511 |
| ENSG00000198899 | 332094.31 | -0.276428108 | 0.07929891 | -3.48590068 | 4.904831e-04 | 2.629533e-03 | MT-ATP6 | protein_coding | 3.870758e-03 | -0.148954602 |
| ENSG00000108821 | 280447.02 | 1.781306396 | 0.10981930 | 16.22034021 | 3.621830e-59 | 2.032531e-55 | COL1A1 | protein_coding | 0.000000e+00 | 0.782937536 |
| ENSG00000184009 | 252331.70 | -0.081625286 | 0.05915722 | -1.37980269 | 1.676474e-01 | 2.638727e-01 | ACTG1 | protein_coding | 6.028432e-03 | 0.141675731 |
| ENSG00000198786 | 232734.28 | -0.269617566 | 0.08432876 | -3.19721963 | 1.387592e-03 | 6.130434e-03 | MT-ND5 | protein_coding | 5.629971e-02 | -0.098664695 |
| ENSG00000156508 | 229425.49 | 0.058731509 | 0.05556939 | 1.05690391 | 2.905554e-01 | 4.028739e-01 | EEF1A1 | protein_coding | 1.302065e-03 | 0.165649107 |
| ENSG00000251562 | 203517.93 | -0.284039979 | 0.12154088 | -2.33699127 | 1.943964e-02 | 4.946037e-02 | MALAT1 | lincRNA | 2.029753e-01 | -0.065877802 |
| ENSG00000019582 | 192317.27 | 0.232000998 | 0.12107859 | 1.91611913 | 5.534993e-02 | 1.116080e-01 | CD74 | protein_coding | 2.806783e-06 | 0.239889180 |
| ENSG00000166710 | 183683.08 | 0.060903215 | 0.08064188 | 0.75523058 | 4.501106e-01 | 5.600822e-01 | B2M | protein_coding | 4.643623e-03 | 0.146001820 |
| ENSG00000211592 | 180268.09 | 0.295200212 | 0.18037165 | 1.63662204 | 1.017095e-01 | 1.789972e-01 | IGKC | IG_C_gene | 3.153365e-04 | 0.185326886 |
| ENSG00000211896 | 172591.58 | 0.630625038 | 0.17992983 | 3.50483874 | 4.568839e-04 | 2.481806e-03 | IGHG1 | IG_C_gene | 6.075227e-07 | 0.255086358 |
| ENSG00000087086 | 169604.28 | 0.103007196 | 0.08009100 | 1.28612700 | 1.983987e-01 | 3.006639e-01 | FTL | protein_coding | 1.516472e-08 | 0.288319490 |
| ENSG00000111640 | 164400.95 | -0.130326549 | 0.07574650 | -1.72056190 | 8.533035e-02 | 1.563441e-01 | GAPDH | protein_coding | 3.628113e-01 | 0.047108203 |
| ENSG00000167658 | 157079.57 | 0.129524564 | 0.06449384 | 2.00832457 | 4.460881e-02 | 9.445197e-02 | EEF2 | protein_coding | 1.963129e-07 | 0.265728069 |
| ENSG00000102837 | 149567.00 | -0.610149521 | 0.33895941 | -1.80006662 | 7.185012e-02 | 1.368996e-01 | OLFM4 | protein_coding | 3.168174e-01 | -0.051830700 |
| ENSG00000168542 | 148897.47 | 1.673486701 | 0.10446214 | 16.02003129 | 9.260366e-58 | 4.251939e-54 | COL3A1 | protein_coding | 0.000000e+00 | 0.780281488 |
| ENSG00000070756 | 145997.67 | -0.160928371 | 0.06830306 | -2.35609316 | 1.846829e-02 | 4.755188e-02 | PABPC1 | protein_coding | 9.482088e-02 | -0.086388668 |
| ENSG00000133112 | 139694.19 | -0.233987579 | 0.06354392 | -3.68229706 | 2.311418e-04 | 1.421266e-03 | TPT1 | protein_coding | 1.106994e-01 | -0.082497668 |
| ENSG00000211895 | 139262.20 | -0.914783674 | 0.21456902 | -4.26335387 | 2.013812e-05 | 1.880610e-04 | IGHA1 | IG_C_gene | 4.868777e-01 | 0.035998407 |
| ENSG00000196924 | 136149.92 | 1.507504713 | 0.14505594 | 10.39257456 | 2.680190e-25 | 4.635903e-23 | FLNA | protein_coding | 0.000000e+00 | 0.622651496 |
| ENSG00000164692 | 134053.88 | 1.668727098 | 0.09955441 | 16.76196019 | 4.631009e-63 | 3.898306e-59 | COL1A2 | protein_coding | 0.000000e+00 | 0.798824667 |
| ENSG00000211459 | 132795.86 | -0.375231559 | 0.09978718 | -3.76031835 | 1.696973e-04 | 1.108927e-03 | MT-RNR1 | Mt_rRNA | 2.597720e-01 | -0.058323586 |
| ENSG00000170421 | 125977.93 | -0.128458200 | 0.10021670 | -1.28180435 | 1.999113e-01 | 3.024427e-01 | KRT8 | protein_coding | 3.845815e-01 | 0.045011492 |
| ENSG00000133392 | 123333.08 | 2.348968643 | 0.23911391 | 9.82363873 | 8.906921e-23 | 1.180740e-20 | MYH11 | protein_coding | 0.000000e+00 | 0.467955626 |
| ENSG00000090382 | 109464.56 | -0.458185680 | 0.17927623 | -2.55575255 | 1.059584e-02 | 3.067371e-02 | LYZ | protein_coding | 9.553314e-01 | -0.002900899 |
| ENSG00000198840 | 107437.13 | -0.246531133 | 0.10421080 | -2.36569648 | 1.799619e-02 | 4.660242e-02 | MT-ND3 | protein_coding | 4.024075e-02 | -0.106003413 |
| ENSG00000115414 | 106327.27 | 1.499856246 | 0.12371174 | 12.12379891 | 7.901006e-34 | 3.117626e-31 | FN1 | protein_coding | 0.000000e+00 | 0.662226875 |
| ENSG00000089157 | 97243.51 | -0.196328853 | 0.06405483 | -3.06501244 | 2.176609e-03 | 8.794721e-03 | RPLP0 | protein_coding | 3.859853e-01 | -0.044878826 |
| ENSG00000162896 | 97177.17 | -0.623813505 | 0.22481680 | -2.77476369 | 5.524183e-03 | 1.834867e-02 | PIGR | protein_coding | 1.573849e-02 | -0.124692684 |
| ENSG00000212907 | 94207.15 | -0.394033750 | 0.07624550 | -5.16796078 | 2.366620e-07 | 4.003043e-06 | MT-ND4L | protein_coding | 3.616474e-05 | -0.212063033 |
| ENSG00000108107 | 93899.45 | -0.184199763 | 0.08213019 | -2.24277769 | 2.491115e-02 | 5.998225e-02 | RPL28 | protein_coding | 2.232663e-01 | 0.063024235 |
| ENSG00000096088 | 91077.14 | -1.123893751 | 0.30725974 | -3.65779698 | 2.543924e-04 | 1.539676e-03 | PGC | protein_coding | 1.583048e-02 | -0.124583684 |
| ENSG00000142541 | 90785.59 | -0.055943925 | 0.06643225 | -0.84211995 | 3.997208e-01 | 5.127810e-01 | RPL13A | protein_coding | 2.224044e-02 | 0.118084424 |
| ENSG00000148346 | 90740.31 | -1.263058776 | 0.21081575 | -5.99129236 | 2.081800e-09 | 5.631788e-08 | LCN2 | protein_coding | 4.052610e-05 | -0.210738878 |
| ENSG00000106541 | 90273.32 | -0.751495081 | 0.15839476 | -4.74444401 | 2.090798e-06 | 2.718845e-05 | AGR2 | protein_coding | 2.657117e-04 | -0.187572875 |
| ENSG00000096384 | 89944.57 | -0.206670280 | 0.06753207 | -3.06032813 | 2.210946e-03 | 8.905675e-03 | HSP90AB1 | protein_coding | 1.236323e-01 | -0.079641825 |
| ENSG00000234745 | 88807.27 | 0.085112019 | 0.09172701 | 0.92788389 | 3.534678e-01 | 4.676761e-01 | HLA-B | protein_coding | 8.737661e-04 | 0.171394243 |
| ENSG00000147403 | 87697.81 | -0.016634576 | 0.06843176 | -0.24308267 | 8.079414e-01 | 8.635972e-01 | RPL10 | protein_coding | 7.516434e-04 | 0.173517351 |
| ENSG00000100345 | 86074.27 | 0.332249579 | 0.05124190 | 6.48394397 | 8.935542e-11 | 3.018779e-09 | MYH9 | protein_coding | 0.000000e+00 | 0.451399477 |
| ENSG00000140988 | 84694.17 | -0.066360176 | 0.07734477 | -0.85797884 | 3.909041e-01 | 5.042627e-01 | RPS2 | protein_coding | 1.699569e-02 | 0.123249061 |
| ENSG00000100316 | 83965.60 | 0.005476985 | 0.06142920 | 0.08915931 | 9.289553e-01 | 9.513706e-01 | RPL3 | protein_coding | 1.663537e-02 | 0.123652975 |
| ENSG00000167526 | 82064.03 | 0.027924175 | 0.07452635 | 0.37468860 | 7.078921e-01 | 7.850026e-01 | RPL13 | protein_coding | 6.304647e-04 | 0.175966777 |
