# Supplementary material for: HTRA3 Is a Prognostic Biomarker and Associated With Immune Infiltrates in Gastric Cancer
Source: Front Oncol. 2020 Dec 23;10:603480. doi: 10.3389/fonc.2020.603480 (PMC7786138; doi:10.3389/fonc.2020.603480)
Supplement: Supplemental Table 1 — Clinical characteristics of gastric cancer patients based on TCGA. [file DataSheet_1.zip › Supplemental Table 3ú║50 items of Gene Ontology enrichment analysis..docx]

Supplemental Table 2: 50 items of Gene ontology (GO) enrichment analysis

| ONTOLOGY | ID | Description | GeneRatio | BgRatio | pvalue | p.adjust | qvalue | geneID | Count |
| --- | --- | --- | --- | --- | --- | --- | --- | --- | --- |
| BP | GO:0030198 | extracellular matrix organization | 41/366 | 368/18670 | 1.613122e-19 | 5.052298e-16 | 4.499761e-16 | COL1A1/COL3A1/COL1A2/MYH11/BGN/COL5A1/MMP2/GREM1/AEBP1/SULF1/TNC/CCDC80/SFRP2/MFAP4/EMILIN1/MMP11/COL8A1/ADAMTS2/FBLN2/DDR2/ITGA11/COL10A1/CYP1B1/COL11A1/ADAM12/MFAP5/COMP/FAP/COL8A2/PRSS1/VTN/TTR/FGB/FGA/FGG/HAS1/CMA1/NR2E1/CTRB2/CTRB1/CAPNS2 | 41 |
| BP | GO:0043062 | extracellular structure organization | 43/366 | 422/18670 | 6.092458e-19 | 9.540789e-16 | 8.497376e-16 | COL1A1/COL3A1/COL1A2/MYH11/BGN/COL5A1/MMP2/GREM1/AEBP1/SULF1/TNC/CCDC80/SFRP2/MFAP4/EMILIN1/MMP11/COL8A1/ADAMTS2/FBLN2/DDR2/ITGA11/COL10A1/CYP1B1/COL11A1/ADAM12/MFAP5/COMP/FAP/COL8A2/PRSS1/VTN/TTR/FGB/FGA/APOA2/FGG/APOC3/HAS1/CMA1/NR2E1/CTRB2/CTRB1/CAPNS2 | 43 |
| BP | GO:0070268 | cornification | 20/366 | 112/18670 | 5.292019e-14 | 5.524867e-11 | 4.920649e-11 | KRT13/KRT6A/KRT4/SPRR3/KRT5/KRT14/SPRR2A/KRT6C/TGM1/SPRR2E/SPRR2D/KRT78/IVL/KRT24/SPRR2F/TGM5/LCE3D/SPRR2G/SPRR2B/KRT33A | 20 |
| BP | GO:0043588 | skin development | 36/366 | 419/18670 | 1.024558e-13 | 8.022290e-11 | 7.144945e-11 | COL1A1/COL3A1/COL1A2/COL5A1/REG3A/ADAMTS2/KRT13/KRT6A/KRT4/COMP/SPRR3/KRT5/S100A7/KRT14/SOSTDC1/SPRR2A/KRT6C/FGF10/TGM3/TGM1/SPRR2E/SPRR2D/REG3G/KRT78/IVL/SERPINB13/CERS3/KRT24/SPRR2F/TGM5/LCE3D/SPRR2G/SPRR2B/LCE3E/KRT33A/KRTAP27-1 | 36 |
| BP | GO:0030199 | collagen fibril organization | 14/366 | 54/18670 | 1.542819e-12 | 9.664215e-10 | 8.607304e-10 | COL1A1/COL3A1/COL1A2/COL5A1/GREM1/AEBP1/SFRP2/EMILIN1/MMP11/ADAMTS2/DDR2/CYP1B1/COL11A1/COMP | 14 |
| BP | GO:0018149 | peptide cross-linking | 14/366 | 60/18670 | 7.413396e-12 | 3.869793e-09 | 3.446579e-09 | COL3A1/BGN/SPRR3/SPRR2A/TGM3/TGM1/SPRR2E/SPRR2D/IVL/SPRR2F/TGM5/LCE3D/SPRR2B/LCE3E | 14 |
| BP | GO:0030216 | keratinocyte differentiation | 28/366 | 305/18670 | 1.324953e-11 | 5.928219e-09 | 5.279889e-09 | REG3A/KRT13/KRT6A/KRT4/SPRR3/KRT5/S100A7/KRT14/SPRR2A/KRT6C/TGM3/TGM1/SPRR2E/SPRR2D/REG3G/KRT78/IVL/SERPINB13/CERS3/KRT24/SPRR2F/TGM5/LCE3D/SPRR2G/SPRR2B/LCE3E/KRT33A/KRTAP27-1 | 28 |
| BP | GO:0009913 | epidermal cell differentiation | 30/366 | 358/18670 | 2.398708e-11 | 9.390940e-09 | 8.363915e-09 | REG3A/SFRP4/KRT13/KRT6A/KRT4/SPRR3/KRT5/S100A7/KRT14/GLI1/SPRR2A/KRT6C/TGM3/TGM1/SPRR2E/SPRR2D/REG3G/KRT78/IVL/SERPINB13/CERS3/KRT24/SPRR2F/TGM5/LCE3D/SPRR2G/SPRR2B/LCE3E/KRT33A/KRTAP27-1 | 30 |
| BP | GO:0008544 | epidermis development | 34/366 | 464/18670 | 4.301159e-11 | 1.496803e-08 | 1.333108e-08 | REG3A/SFRP4/KRT13/KRT6A/KRT4/SPRR3/KRT5/S100A7/KRT14/SOSTDC1/GLI1/SPRR2A/KRT6C/FGF10/TGM3/TGM1/SPRR2E/SPRR2D/REG3G/KRT78/IVL/SERPINB13/CERS3/KRTDAP/CALML5/KRT24/SPRR2F/TGM5/LCE3D/SPRR2G/SPRR2B/LCE3E/KRT33A/KRTAP27-1 | 34 |
| BP | GO:0031424 | keratinization | 23/366 | 224/18670 | 1.007621e-10 | 3.155870e-08 | 2.810733e-08 | KRT13/KRT6A/KRT4/SPRR3/KRT5/KRT14/SPRR2A/KRT6C/TGM3/TGM1/SPRR2E/SPRR2D/KRT78/IVL/KRT24/SPRR2F/TGM5/LCE3D/SPRR2G/SPRR2B/LCE3E/KRT33A/KRTAP27-1 | 23 |
| BP | GO:0006936 | muscle contraction | 27/366 | 360/18670 | 2.845373e-09 | 8.101553e-07 | 7.215539e-07 | FLNA/MYH11/DES/ACTG2/MYL9/ACTA2/MYLK/CNN1/SULF1/SYNM/HSPB6/LMOD1/KCNMA1/PLN/SGCD/COMP/TACR2/ATP1A2/CHRM2/CASQ2/CHRNA3/SCN7A/SMPX/KCNA5/NKX2-5/KCNA1/SCN2B | 27 |
| BP | GO:0001503 | ossification | 27/366 | 398/18670 | 2.393060e-08 | 6.245888e-06 | 5.562816e-06 | COL1A1/COL1A2/MMP2/GREM1/TNC/MGP/SFRP2/CDH11/ASPN/DDR2/ITGA11/CHRDL2/CTHRC1/COL11A1/COMP/SFRP1/HAND2/CHRDL1/OMD/GLI1/BMP3/TWIST2/TNN/PENK/TAC1/GDF10/STATH | 27 |
| BP | GO:0003012 | muscle system process | 29/366 | 465/18670 | 4.528440e-08 | 1.091006e-05 | 9.716896e-06 | FLNA/MYH11/DES/ACTG2/MYL9/ACTA2/MYLK/CNN1/SULF1/SYNM/HSPB6/LMOD1/KCNMA1/PLN/SGCD/COMP/TACR2/HAND2/ATP1A2/CHRM2/PI16/CASQ2/CHRNA3/SCN7A/SMPX/KCNA5/NKX2-5/KCNA1/SCN2B | 29 |
| BP | GO:0007178 | transmembrane receptor protein serine/threonine kinase signaling pathway | 23/366 | 349/18670 | 4.464844e-07 | 9.988495e-05 | 8.896119e-05 | COL3A1/COL1A2/GREM1/SULF1/SFRP2/EMILIN1/SFRP4/HTRA3/ASPN/RGMA/COMP/SFRP1/CILP/CHRDL1/SOSTDC1/BMP3/FGF10/NKX2-5/GDF10/AFP/GDF6/CIDEA/IL17F | 23 |
| BP | GO:0030007 | cellular potassium ion homeostasis | 5/366 | 13/18670 | 3.185743e-06 | 6.651831e-04 | 5.924364e-04 | KCNMA1/ATP1A2/ATP4A/ATP4B/ATP12A | 5 |
| BP | GO:0007586 | digestion | 13/366 | 139/18670 | 3.591807e-06 | 7.030962e-04 | 6.262031e-04 | GKN1/PRSS1/SLC9A4/NPR3/PGA5/APOA2/FGF10/TAC1/PGA4/CHIA/CTRB2/CTRB1/STATH | 13 |
| BP | GO:0006813 | potassium ion transport | 17/366 | 240/18670 | 5.567292e-06 | 9.890409e-04 | 8.808760e-04 | FLNA/FHL1/KCNMA1/KCNMB1/ATP1A2/SLC9A4/KCNK3/CASQ2/ATP4A/VIP/KCNB1/ATP4B/NALCN/DPP6/KCNA5/KCNA1/ATP12A | 17 |
| BP | GO:0090092 | regulation of transmembrane receptor protein serine/threonine kinase signaling pathway | 17/366 | 241/18670 | 5.884419e-06 | 9.890409e-04 | 8.808760e-04 | GREM1/SULF1/SFRP2/EMILIN1/SFRP4/HTRA3/ASPN/SFRP1/CILP/CHRDL1/SOSTDC1/BMP3/FGF10/GDF10/GDF6/CIDEA/IL17F | 17 |
| BP | GO:0071804 | cellular potassium ion transport | 16/366 | 217/18670 | 6.315714e-06 | 9.890409e-04 | 8.808760e-04 | FLNA/FHL1/KCNMA1/KCNMB1/ATP1A2/SLC9A4/KCNK3/CASQ2/ATP4A/KCNB1/ATP4B/NALCN/DPP6/KCNA5/KCNA1/ATP12A | 16 |
| BP | GO:0071805 | potassium ion transmembrane transport | 16/366 | 217/18670 | 6.315714e-06 | 9.890409e-04 | 8.808760e-04 | FLNA/FHL1/KCNMA1/KCNMB1/ATP1A2/SLC9A4/KCNK3/CASQ2/ATP4A/KCNB1/ATP4B/NALCN/DPP6/KCNA5/KCNA1/ATP12A | 16 |
| BP | GO:0055078 | sodium ion homeostasis | 8/366 | 52/18670 | 7.162024e-06 | 1.068165e-03 | 9.513466e-04 | C7/ATP1A2/ATP4A/SCN7A/ATP4B/SCNN1G/TAC1/ATP12A | 8 |
| BP | GO:0003018 | vascular process in circulatory system | 14/366 | 173/18670 | 8.320788e-06 | 1.184578e-03 | 1.055028e-03 | ACTA2/KCNMA1/KCNMB1/COMP/TACR2/SLIT2/ATP1A2/FGB/FGA/VIP/FGG/KCNA5/ADRB3/CRP | 14 |
| BP | GO:0006939 | smooth muscle contraction | 11/366 | 110/18670 | 1.065594e-05 | 1.451061e-03 | 1.292368e-03 | MYH11/ACTA2/MYLK/CNN1/SULF1/KCNMA1/COMP/TACR2/ATP1A2/CHRM2/CHRNA3 | 11 |
| BP | GO:0030278 | regulation of ossification | 15/366 | 203/18670 | 1.196961e-05 | 1.562034e-03 | 1.391204e-03 | GREM1/MGP/SFRP2/DDR2/CTHRC1/COMP/SFRP1/HAND2/OMD/GLI1/TWIST2/TNN/TAC1/GDF10/STATH | 15 |
| BP | GO:0006883 | cellular sodium ion homeostasis | 5/366 | 17/18670 | 1.435726e-05 | 1.798677e-03 | 1.601968e-03 | C7/ATP1A2/ATP4A/ATP4B/ATP12A | 5 |
| BP | GO:0055075 | potassium ion homeostasis | 6/366 | 29/18670 | 1.767031e-05 | 2.128592e-03 | 1.895802e-03 | KCNMA1/ATP1A2/ATP4A/ATP4B/KCNA5/ATP12A | 6 |
| BP | GO:0035296 | regulation of tube diameter | 12/366 | 143/18670 | 2.554900e-05 | 2.719904e-03 | 2.422446e-03 | ACTA2/KCNMA1/KCNMB1/COMP/ATP1A2/FGB/FGA/VIP/FGG/KCNA5/ADRB3/CRP | 12 |
| BP | GO:0050880 | regulation of blood vessel size | 12/366 | 143/18670 | 2.554900e-05 | 2.719904e-03 | 2.422446e-03 | ACTA2/KCNMA1/KCNMB1/COMP/ATP1A2/FGB/FGA/VIP/FGG/KCNA5/ADRB3/CRP | 12 |
| BP | GO:0097746 | regulation of blood vessel diameter | 12/366 | 143/18670 | 2.554900e-05 | 2.719904e-03 | 2.422446e-03 | ACTA2/KCNMA1/KCNMB1/COMP/ATP1A2/FGB/FGA/VIP/FGG/KCNA5/ADRB3/CRP | 12 |
| BP | GO:0022617 | extracellular matrix disassembly | 9/366 | 80/18670 | 2.637812e-05 | 2.719904e-03 | 2.422446e-03 | MMP2/MMP11/DDR2/FAP/PRSS1/CMA1/CTRB2/CTRB1/CAPNS2 | 9 |
| BP | GO:0035150 | regulation of tube size | 12/366 | 144/18670 | 2.738832e-05 | 2.719904e-03 | 2.422446e-03 | ACTA2/KCNMA1/KCNMB1/COMP/ATP1A2/FGB/FGA/VIP/FGG/KCNA5/ADRB3/CRP | 12 |
| BP | GO:0060346 | bone trabecula formation | 4/366 | 10/18670 | 2.778957e-05 | 2.719904e-03 | 2.422446e-03 | COL1A1/MMP2/GREM1/SFRP1 | 4 |
| BP | GO:0006937 | regulation of muscle contraction | 13/366 | 171/18670 | 3.381030e-05 | 3.208904e-03 | 2.857968e-03 | FLNA/MYL9/CNN1/HSPB6/KCNMA1/PLN/TACR2/ATP1A2/CHRM2/CASQ2/CHRNA3/NKX2-5/KCNA1 | 13 |
| BP | GO:0001649 | osteoblast differentiation | 15/366 | 225/18670 | 4.005214e-05 | 3.689509e-03 | 3.286012e-03 | COL1A1/GREM1/TNC/SFRP2/DDR2/ITGA11/CTHRC1/SFRP1/HAND2/GLI1/BMP3/TWIST2/TNN/PENK/GDF10 | 15 |
| BP | GO:0055067 | monovalent inorganic cation homeostasis | 12/366 | 154/18670 | 5.310899e-05 | 4.752496e-03 | 4.232747e-03 | C7/KCNMA1/ATP1A2/SLC9A4/RHCG/ATP4A/SCN7A/ATP4B/KCNA5/SCNN1G/TAC1/ATP12A | 12 |
| BP | GO:0042340 | keratan sulfate catabolic process | 4/366 | 12/18670 | 6.349523e-05 | 5.423931e-03 | 4.830751e-03 | PRELP/OGN/OMD/KERA | 4 |
| BP | GO:0030509 | BMP signaling pathway | 12/366 | 157/18670 | 6.407581e-05 | 5.423931e-03 | 4.830751e-03 | GREM1/SULF1/SFRP2/SFRP4/HTRA3/RGMA/COMP/SFRP1/CHRDL1/SOSTDC1/NKX2-5/GDF6 | 12 |
| BP | GO:0051216 | cartilage development | 14/366 | 209/18670 | 6.846851e-05 | 5.643247e-03 | 5.026082e-03 | COL1A1/GREM1/SULF1/MGP/SFRP2/CHRDL2/PRRX1/COL11A1/COMP/HAND2/BMP3/NKX3-2/GDF6/IL17F | 14 |
| BP | GO:0060343 | trabecula formation | 5/366 | 24/18670 | 8.808012e-05 | 6.897996e-03 | 6.143608e-03 | COL1A1/MMP2/GREM1/SFRP1/NKX2-5 | 5 |
| BP | GO:0010838 | positive regulation of keratinocyte proliferation | 4/366 | 13/18670 | 9.029944e-05 | 6.897996e-03 | 6.143608e-03 | REG3A/FGF10/TGM1/REG3G | 4 |
| BP | GO:0061430 | bone trabecula morphogenesis | 4/366 | 13/18670 | 9.029944e-05 | 6.897996e-03 | 6.143608e-03 | COL1A1/MMP2/GREM1/SFRP1 | 4 |
| BP | GO:0061448 | connective tissue development | 16/366 | 273/18670 | 1.039349e-04 | 7.750577e-03 | 6.902948e-03 | COL1A1/ACTA2/COL5A1/GREM1/SULF1/MGP/SFRP2/CHRDL2/PRRX1/COL11A1/COMP/HAND2/BMP3/NKX3-2/GDF6/IL17F | 16 |
| BP | GO:0010248 | establishment or maintenance of transmembrane electrochemical gradient | 4/366 | 14/18670 | 1.244688e-04 | 8.859917e-03 | 7.890966e-03 | ATP1A2/ATP4A/ATP4B/ATP12A | 4 |
| BP | GO:0036376 | sodium ion export across plasma membrane | 4/366 | 14/18670 | 1.244688e-04 | 8.859917e-03 | 7.890966e-03 | ATP1A2/ATP4A/ATP4B/ATP12A | 4 |
| BP | GO:1902041 | regulation of extrinsic apoptotic signaling pathway via death domain receptors | 7/366 | 58/18670 | 1.337106e-04 | 8.952852e-03 | 7.973737e-03 | SFRP2/TIMP3/SFRP1/FGB/FGA/MAL/FGG | 7 |
| BP | GO:0010811 | positive regulation of cell-substrate adhesion | 10/366 | 121/18670 | 1.363386e-04 | 8.952852e-03 | 7.973737e-03 | FLNA/CCDC80/EMILIN1/COL8A1/FBLN2/SFRP1/VTN/FGB/FGA/FGG | 10 |
| BP | GO:0071772 | response to BMP | 12/366 | 170/18670 | 1.372085e-04 | 8.952852e-03 | 7.973737e-03 | GREM1/SULF1/SFRP2/SFRP4/HTRA3/RGMA/COMP/SFRP1/CHRDL1/SOSTDC1/NKX2-5/GDF6 | 12 |
| BP | GO:0071773 | cellular response to BMP stimulus | 12/366 | 170/18670 | 1.372085e-04 | 8.952852e-03 | 7.973737e-03 | GREM1/SULF1/SFRP2/SFRP4/HTRA3/RGMA/COMP/SFRP1/CHRDL1/SOSTDC1/NKX2-5/GDF6 | 12 |
| BP | GO:0046851 | negative regulation of bone remodeling | 4/366 | 15/18670 | 1.671133e-04 | 1.068161e-02 | 9.513430e-03 | GREM1/SFRP1/CARTPT/CALCA | 4 |
| BP | GO:0045667 | regulation of osteoblast differentiation | 10/366 | 126/18670 | 1.904448e-04 | 1.122488e-02 | 9.997287e-03 | GREM1/SFRP2/DDR2/CTHRC1/SFRP1/HAND2/GLI1/TWIST2/TNN/GDF10 | 10 |
