# Supplementary material for: HTRA3 Is a Prognostic Biomarker and Associated With Immune Infiltrates in Gastric Cancer
Source: Front Oncol. 2020 Dec 23;10:603480. doi: 10.3389/fonc.2020.603480 (PMC7786138; doi:10.3389/fonc.2020.603480)
Supplement: Supplemental Table 1 — Clinical characteristics of gastric cancer patients based on TCGA. [file DataSheet_1.zip › Supplemental Table 4ú║Gene sets enriched in phenotype high..docx]

| ID | Set  Size | Enrichment Score | NES | pvalue | | p.adjust | FDR | rank | leading_edge | core_enrichment |
| --- | --- | --- | --- | --- | --- | --- | --- | --- | --- | --- |
| KEGG_OLFACTORY_TRANSDUCTION | 355 | -0.514 | -1.943 | 0.001 | | 0.033 | 0.020 | 10693 | tags=38%, list=21%, signal=30% | OR2M4/OR51I2/OR52A5/OR8U1/OR2H1/OR1M1/OR56B4/OR10AG1/OR10A3/OR1A2/OR52N2/OR2H2/OR6C3/OR2K2/OR4P4/OR13H1/OR10G3/OR2T3/OR52N4/OR5B21/OR4B1/OR10V1/OR6B2/OR2B2/OR2D3/OR2A4/GUCA1C/CALML6/OR1Q1/CNGB1/OR2AT4/OR13F1/OR9A4/OR2T10/OR2G3/OR1E1/OR6Y1/OR7G3/OR52J3/PRKACG/OR51V1/OR10G2/OR10A2/OR4D10/OR3A3/OR4C12/OR6C68/OR2V2/OR52B2/OR51B4/OR9Q1/OR10AD1/OR2Y1/OR52E2/OR7D4/OR6C6/OR4N2/OR1J1/OR7A10/OR52E4/OR4K13/OR52N5/OR8S1/OR51D1/OR13C8/OR1L3/OR1G1/OR11H4/OR11G2/OR5M8/OR2B6/OR10P1/OR5B12/OR8D4/OR10K1/OR2G6/OR56A3/OR5C1/OR6S1/OR2T34/OR6K2/OR13D1/OR10H3/OR5AC2/OR1L1/OR8B12/OR2M7/OR4X2/OR2G2/OR7A17/OR5J2/OR2F2/OR4K5/OR6M1/OR10J5/CLCA1/OR5T3/OR4D9/OR5M11/OR6N2/OR2B3/OR7C2/OR5AP2/OR12D3/OR8K1/OR4K2/OR2L2/OR10H2/OR11L1/OR8J1/OR9A2/OR4D11/OR5AU1/OR5A1/OR4D1/OR11A1/OR1A1/OR4K14/OR52E6/OR52A1/OR4D5/OR51Q1/OR8K5/OR10J3/OR6C75/OR6C2/OR10K2/OR6C65/CALML5/OR8H2/OR51A7/CLCA2/CALML3/CLCA4 |
| REACTOME_OLFACTORY_SIGNALING_PATHWAY | 361 | -0.503 | -1.900 | 0.001 | | 0.033 | 0.020 | 10693 | tags=37%, list=21%, signal=29% | OR2M4/OR51I2/OR52A5/OR8U1/OR2H1/OR1M1/OR56B4/OR10AG1/OR10A3/OR6J1/OR1A2/OR52N2/OR2H2/OR6C3/OR2K2/OR4P4/GNGT1/OR13H1/OR10G3/OR2T3/OR52N4/OR5B21/OR4B1/OR10V1/OR6B2/OR2B2/OR2D3/OR2A4/OR10D3/OR1Q1/OR2AT4/OR13F1/OR9A4/OR2T10/OR2G3/OR1E1/OR6Y1/OR7G3/OR52J3/OR51V1/OR10G2/OR10A2/OR5W2/OR4D10/OR3A3/OR4C12/OR6C68/OR2V2/OR52B2/OR51B4/OR9Q1/OR10AD1/OR11H2/OR2Y1/OR52E2/OR7D4/OR6C6/OR4N2/OR1J1/OR7A10/OR52E4/OR4K13/OR52N5/OR8S1/OR51D1/OR13C8/OR1L3/OR1G1/OR11H4/OR11G2/OR5M8/OR10W1/OR2B6/OR10P1/OR5B12/OR8D4/OR10K1/OR2G6/OR56A3/OR5C1/OR6S1/OR2T34/OR6K2/OR13D1/OR10H3/OR5AC2/OR1L1/OR8B12/OR4C5/OR2M7/OR4X2/OR2G2/OR7A17/OR5J2/OR2F2/OR4K5/OR6M1/OR10J5/OR5T3/OR4D9/OR5M11/OR6N2/OR2B3/OR7C2/OR5AP2/OR12D3/OR8K1/OR4K2/OR2L2/OR10H2/OR11L1/OR8J1/OR9A2/OR4D11/OR5AU1/OR5A1/OR4D1/OR11A1/OR1A1/OR4K14/OR52E6/OR52A1/OR4D5/OR51Q1/OR8K5/OR10J3/OR6C75/OR6C2/OR10K2/OR6C65/OR8H2/OR51A7 |
| REACTOME_KERATINIZATION | 215 | -0.642 | -2.362 | 0.001 | | 0.033 | 0.020 | 8650 | tags=46%, list=17%, signal=38% | KRTAP1-5/KRTAP12-3/DSC2/KRTAP20-2/KRTAP5-4/KRTAP10-5/KRTAP9-6/SPINK9/LCE1C/LIPK/KRTAP5-9/KRT23/KRTAP19-7/KRTAP6-3/KRTAP10-8/KRT82/KRTAP16-1/KRTAP10-6/KRTAP4-4/KRTAP17-1/CASP14/KRTAP10-11/KRTAP9-1/KRTAP12-1/KRTAP5-5/KRT77/KRTAP5-8/KRTAP13-2/KRTAP1-1/KRTAP12-4/KRT15/KLK13/KRTAP29-1/KRT31/KLK12/KRTAP3-2/KRT2/DSG3/KRTAP9-3/LCE2C/LELP1/KRT32/KRT73/KRT25/KRTAP21-3/SPINK6/KRTAP4-6/LCE1A/KRTAP10-2/SPINK5/LCE1B/KRTAP5-10/KRT72/KRTAP9-4/KRTAP6-2/PI3/KRT28/KRTAP5-3/KRTAP9-9/KRTAP4-8/RPTN/KRTAP26-1/KRT16/DSG1/KRTAP13-1/KRTAP3-1/KRTAP1-3/KRT75/CELA2A/KRTAP11-1/LCE6A/SPRR1A/KRTAP19-5/KRT6B/PKP1/CSTA/TGM5/KRTAP27-1/SPRR2D/LCE3D/TGM1/KRT33A/KRT6A/SPRR2A/KRT24/SPRR3/SPRR2G/SPRR2F/LCE3E/KRT78/KRT14/KRT5/IVL/SPRR2E/SPRR2B/KRT6C/KRT13/KRT4 |
| REACTOME_FORMATION_OF_THE_CORNIFIED_ENVELOPE | 128 | -0.683 | -2.398 | | 0.001 | 0.033 | 0.020 | 4884 | tags=38%, list=9%, signal=35% | KRT15/KLK13/KRT31/KLK12/KRT2/DSG3/LCE2C/LELP1/KRT32/KRT73/KRT25/SPINK6/LCE1A/SPINK5/LCE1B/KRT72/PI3/KRT28/RPTN/KRT16/DSG1/KRT75/CELA2A/LCE6A/SPRR1A/KRT6B/PKP1/CSTA/TGM5/SPRR2D/LCE3D/TGM1/KRT33A/KRT6A/SPRR2A/KRT24/SPRR3/SPRR2G/SPRR2F/LCE3E/KRT78/KRT14/KRT5/IVL/SPRR2E/SPRR2B/KRT6C/KRT13/KRT4 |
| REACTOME_ANTIMICROBIAL_PEPTIDES | 92 | -0.567 | -1.932 | 0.001 | | 0.033 | 0.020 | 7139 | tags=39%, list=14%, signal=34% | DEFB127/LTF/HTN1/CHGA/RNASE7/CAMP/DEFB132/DEFA6/EPPIN-WFDC6/DEFB108B/PGLYRP4/DCD/BPIFB6/DEFA4/HTN3/DEFB116/DEFB126/PI3/DEFA5/DEFB118/BPIFB1/DEFB123/S100A9/DEFB4B/DEFB1/BPIFB2/BPIFA2/LCN2/PGLYRP3/S100A8/ITLN1/S100A7A/REG3A/DEFB4A/S100A7/REG3G |
| REACTOME_DIGESTION_AND_ABSORPTION | 27 | -0.663 | -1.845 | 0.001 | | 0.033 | 0.020 | 3672 | tags=44%, list=7%, signal=41% | PNLIPRP1/ALPI/GUCA2A/LCT/GUCA2B/PNLIPRP3/PNLIP/SLC2A2/CEL/PNLIPRP2/LIPF/CHIA |
| REACTOME_DIGESTION | 23 | -0.683 | -1.831 | 0.001 | | 0.033 | 0.020 | 3672 | tags=57%, list=7%, signal=53% | CLPS/MGAM/PNLIPRP1/ALPI/GUCA2A/LCT/GUCA2B/PNLIPRP3/PNLIP/CEL/PNLIPRP2/LIPF/CHIA |
| REACTOME_TYPE_I_HEMIDESMOSOME_ASSEMBLY | 11 | -0.785 | -1.789 | 0.001 | | 0.033 | 0.020 | 16 | tags=18%, list=0%, signal=18% | KRT14/KRT5 |
| REACTOME_CHYLOMICRON_ASSEMBLY | 10 | -0.777 | -1.722 | 0.001 | | 0.033 | 0.020 | 1346 | tags=50%, list=3%, signal=49% | APOB/APOA4/APOA1/APOA2/APOC3 |
| REACTOME_G_ALPHA_S_SIGNALLING_EVENTS | 499 | -0.369 | -1.402 | 0.002 | | 0.033 | 0.020 | 10693 | tags=31%, list=21%, signal=25% | OR2M4/OR51I2/OR52A5/OR8U1/OR2H1/OR1M1/OR56B4/OR10AG1/OR10A3/OR6J1/OR1A2/PTH/OR52N2/OR2H2/OR6C3/OR2K2/OR4P4/RXFP2/GNGT1/OR13H1/OR10G3/OR2T3/OR52N4/OR5B21/OR4B1/HTR4/OR10V1/GPR32/OR6B2/VIPR1/OR2B2/OR2D3/OR2A4/OR10D3/OR1Q1/GIPR/OR2AT4/OR13F1/TAAR2/OR9A4/OR2T10/OR2G3/OR1E1/OR6Y1/IAPP/OR7G3/OR52J3/OR51V1/OR10G2/OR10A2/OR5W2/OR4D10/OR3A3/OR4C12/OR6C68/OR2V2/OR52B2/OR51B4/OR9Q1/OR10AD1/OR11H2/OR2Y1/OR52E2/OR7D4/OR6C6/OR4N2/OR1J1/OR7A10/OR52E4/OR4K13/OR52N5/OR8S1/OR51D1/OR13C8/TAAR8/OR1L3/OR1G1/OR11H4/OR11G2/AVP/OR5M8/PTH2R/PDE11A/GPHA2/RLN2/OR10W1/FSHB/RLN3/OR2B6/OR10P1/OR5B12/OR8D4/OR10K1/OR2G6/OR56A3/OR5C1/OR6S1/OR2T34/OR6K2/OR13D1/OR10H3/OR5AC2/OR1L1/OR8B12/OR4C5/OR2M7/OR4X2/OR2G2/OR7A17/OR5J2/OR2F2/OR4K5/OR6M1/OR10J5/OR5T3/OR4D9/OR5M11/OR6N2/OR2B3/GIP/OR7C2/OR5AP2/OR12D3/OR8K1/OR4K2/OR2L2/OR10H2/OR11L1/OR8J1/OR9A2/OR4D11/OR5AU1/OR5A1/OR4D1/OR11A1/OR1A1/OR4K14/OR52E6/OR52A1/OR4D5/OR51Q1/OR8K5/OR10J3/OR6C75/OR6C2/GPHB5/OR10K2/OR6C65/OR8H2/TAAR5/OR51A7/CGA/CALCA |
| KEGG_METABOLISM_OF_XENOBIOTICS_BY_CYTOCHROME_P450 | 69 | -0.497 | -1.638 | 0.002 | | 0.033 | 0.020 | 10769 | tags=49%, list=21%, signal=39% | GSTO2/GSTA4/GSTM1/UGT2B28/ADH1C/CYP2B6/CYP1A2/CYP2C8/UGT1A10/CYP3A5/AKR1C3/CYP2C18/CYP2C19/UGT2B15/UGT1A6/AKR1C4/UGT1A5/GSTA1/CYP2F1/UGT1A9/CYP3A43/AKR1C2/GSTA5/CYP2C9/UGT2B4/UGT1A8/ALDH3B2/UGT1A1/ALDH3A1/GSTA2/UGT1A7/UGT2A1/GSTA3/ADH7 |
| REACTOME_O2_CO2_EXCHANGE_IN_ERYTHROCYTES | 13 | -0.714 | -1.703 | 0.003 | | 0.033 | 0.020 | 5902 | tags=62%, list=11%, signal=55% | HBB/HBA2/RHAG/HBA1/SLC4A1/CA2/CA1/CA4 |
| BIOCARTA_NPP1_PATHWAY | 10 | 0.871 | 2.245 | 0.003 | | 0.033 | 0.020 | 6709 | tags=100%, list=13%, signal=87% | SPP1/IBSP/COL4A6/COL4A2/COL4A4/COL4A1/COL4A5/COL4A3/ENPP1/ALPL |
| REACTOME_ADENYLATE_CYCLASE_ACTIVATING_PATHWAY | 10 | 0.814 | 2.097 | 0.003 | | 0.033 | 0.020 | 4792 | tags=60%, list=9%, signal=54% | ADCY2/ADCY5/GNAL/ADCY9/ADCY4/ADCY7 |
| REACTOME_NEUROTOXICITY_OF_CLOSTRIDIUM_TOXINS | 10 | 0.829 | 2.136 | 0.003 | | 0.033 | 0.020 | 6480 | tags=70%, list=12%, signal=61% | SNAP25/SV2B/SV2A/SV2C/STX1B/SYT1/VAMP2 |
| REACTOME_P75NTR_REGULATES_AXONOGENESIS | 10 | 0.801 | 2.065 | 0.003 | | 0.033 | 0.020 | 7572 | tags=60%, list=15%, signal=51% | NGFR/NGF/MAG/RTN4/MCF2/LINGO1 |
| REACTOME_REGULATION_OF_COMMISSURAL_AXON_PATHFINDING_BY_SLIT_AND_ROBO | 10 | 0.802 | 2.065 | 0.003 | | 0.033 | 0.020 | 5335 | tags=60%, list=10%, signal=54% | SLIT2/SLIT3/NTN1/ROBO1/ROBO2/SLIT1 |
| BIOCARTA_CELL2CELL_PATHWAY | 12 | 0.774 | 2.074 | 0.004 | | 0.033 | 0.020 | 2762 | tags=42%, list=5%, signal=39% | CTNNA3/ACTN2/ACTN1/PECAM1/VCL |
| BIOCARTA_TCRA_PATHWAY | 12 | 0.815 | 2.184 | 0.004 | | 0.033 | 0.020 | 9346 | tags=92%, list=18%, signal=75% | CD4/FYN/HLA-DRB1/CD3E/LCK/CD3G/CD247/HLA-DRA/ZAP70/CD3D/HLA-DRB5 |
| BIOCARTA_TCYTOTOXIC_PATHWAY | 12 | 0.820 | 2.200 | 0.004 | | 0.033 | 0.020 | 9151 | tags=92%, list=18%, signal=76% | THY1/ITGB2/CD8A/ICAM1/CD3E/CD2/CD3G/CD247/ITGAL/CD28/CD3D |
| BIOCARTA_THELPER_PATHWAY | 12 | 0.821 | 2.200 | 0.004 | | 0.033 | 0.020 | 9151 | tags=92%, list=18%, signal=76% | THY1/ITGB2/CD4/ICAM1/CD3E/CD2/CD3G/CD247/ITGAL/CD28/CD3D |
| REACTOME_PHYSIOLOGICAL_FACTORS | 12 | 0.832 | 2.232 | 0.004 | | 0.033 | 0.020 | 2592 | tags=50%, list=5%, signal=48% | NKX2-5/CORIN/NPR1/TBX5/WWTR1/NPR2 |
| BIOCARTA_MONOCYTE_PATHWAY | 11 | 0.785 | 2.087 | 0.004 | | 0.033 | 0.020 | 7408 | tags=82%, list=14%, signal=70% | SELP/ITGB2/SELE/PECAM1/ITGAM/ITGB1/ICAM1/ITGA4/ITGAL |
| REACTOME_DERMATAN_SULFATE_BIOSYNTHESIS | 11 | 0.900 | 2.395 | 0.004 | | 0.033 | 0.020 | 3945 | tags=64%, list=8%, signal=59% | BGN/DCN/VCAN/CSPG4/DSEL/DSE/CHST14 |
| REACTOME_GP1B_IX_V_ACTIVATION_SIGNALLING | 11 | 0.805 | 2.141 | 0.004 | | 0.033 | 0.020 | 5592 | tags=55%, list=11%, signal=49% | COL1A1/COL1A2/FLNA/VWF/GP5/PIK3R1 |
| REACTOME_IRAK4_DEFICIENCY_TLR2_4 | 11 | 0.772 | 2.054 | 0.004 | | 0.033 | 0.020 | 7648 | tags=64%, list=15%, signal=54% | CD14/CD36/LY96/TLR4/TLR1/TLR2/TLR6 |
| REACTOME_SIGNALING_BY_LEPTIN | 11 | 0.813 | 2.163 | 0.004 | | 0.033 | 0.020 | 5448 | tags=64%, list=10%, signal=57% | LEP/LEPR/IRS1/SOCS3/STAT5A/IRS2/STAT5B |
| REACTOME_SODIUM_CALCIUM_EXCHANGERS | 11 | 0.851 | 2.265 | 0.004 | | 0.033 | 0.020 | 4220 | tags=55%, list=8%, signal=50% | SLC8A2/SLC24A2/SLC24A3/SLC8A1/SLC24A5/SLC8A3 |
| BIOCARTA_ACE2_PATHWAY | 13 | 0.742 | 2.031 | 0.004 | | 0.033 | 0.020 | 3805 | tags=62%, list=7%, signal=57% | CMA1/AGTR2/AGTR1/COL4A6/COL4A2/COL4A4/COL4A1/COL4A5 |
| BIOCARTA_ASBCELL_PATHWAY | 13 | 0.700 | 1.917 | 0.004 | | 0.033 | 0.020 | 9346 | tags=69%, list=18%, signal=57% | IL10/CD4/HLA-DRB1/HLA-DRA/FASLG/CD40/FAS/CD28/HLA-DRB5 |
| BIOCARTA_BARR_MAPK_PATHWAY | 13 | 0.755 | 2.068 | 0.004 | | 0.033 | 0.020 | 8485 | tags=38%, list=16%, signal=32% | KCNA1/KCNA2/DNM1/KCNA3/ARRB1 |
| BIOCARTA_CTL_PATHWAY | 13 | 0.789 | 2.160 | 0.004 | | 0.033 | 0.020 | 9151 | tags=85%, list=18%, signal=70% | ITGB2/GZMB/ICAM1/CD3E/CD3G/CD247/PRF1/FASLG/ITGAL/FAS/CD3D |
| BIOCARTA_LYM_PATHWAY | 13 | 0.711 | 1.947 | 0.004 | | 0.033 | 0.020 | 7408 | tags=69%, list=14%, signal=59% | ITGB2/CD34/PECAM1/ICAM2/VCAM1/ITGB1/ICAM1/ITGA4/ITGAL |
| REACTOME_KERATAN_SULFATE_DEGRADATION | 13 | 0.865 | 2.367 | 0.004 | | 0.033 | 0.020 | 353 | tags=38%, list=1%, signal=38% | KERA/PRELP/OMD/OGN/LUM |
| REACTOME_NF_KB_IS_ACTIVATED_AND_SIGNALS_SURVIVAL | 13 | 0.708 | 1.939 | 0.004 | | 0.033 | 0.020 | 1111 | tags=15%, list=2%, signal=15% | NGFR/NGF |
| REACTOME_P75NTR_RECRUITS_SIGNALLING_COMPLEXES | 13 | 0.709 | 1.942 | 0.004 | | 0.033 | 0.020 | 1111 | tags=15%, list=2%, signal=15% | NGFR/NGF |
| REACTOME_TRAFFICKING_AND_PROCESSING_OF_ENDOSOMAL_TLR | 13 | 0.714 | 1.955 | 0.004 | | 0.033 | 0.020 | 6704 | tags=46%, list=13%, signal=40% | CTSK/TLR7/CTSB/TLR8/CTSL/LGMN |
| BIOCARTA_CLASSIC_PATHWAY | 14 | 0.765 | 2.135 | 0.004 | | 0.033 | 0.020 | 2066 | tags=64%, list=4%, signal=62% | C7/C1R/C1S/C4A/C4B/C3/C1QC/C1QA/C1QB |
| BIOCARTA_ERYTH_PATHWAY | 14 | 0.687 | 1.917 | 0.004 | | 0.033 | 0.020 | 1815 | tags=43%, list=3%, signal=41% | TGFB3/IGF1/TGFB2/IL6/IL11/TGFB1 |
| BIOCARTA_GRANULOCYTES_PATHWAY | 14 | 0.749 | 2.092 | 0.004 | | 0.033 | 0.020 | 7408 | tags=71%, list=14%, signal=61% | SELP/ITGB2/PECAM1/ICAM2/SELPLG/ITGAM/ICAM1/IFNG/TNF/ITGAL |
| PID_S1P_S1P4_PATHWAY | 14 | 0.774 | 2.160 | 0.004 | | 0.033 | 0.020 | 8880 | tags=50%, list=17%, signal=41% | GNAO1/GNAZ/GNAI2/S1PR4/GNA12/GNAI1/PLCG1 |
| REACTOME_ADENYLATE_CYCLASE_INHIBITORY_PATHWAY | 14 | 0.721 | 2.012 | 0.004 | | 0.033 | 0.020 | 4792 | tags=50%, list=9%, signal=45% | ADCY2/ADCY5/GNAL/ADCY9/GNAI2/ADCY4/ADCY7 |
| REACTOME_CS_DS_DEGRADATION | 14 | 0.737 | 2.056 | 0.004 | | 0.033 | 0.020 | 4028 | tags=43%, list=8%, signal=40% | BGN/DCN/VCAN/CSPG4/ARSB/IDS |
| REACTOME_DEFECTIVE_EXT2_CAUSES_EXOSTOSES_2 | 14 | 0.750 | 2.093 | 0.004 | | 0.033 | 0.020 | 4103 | tags=36%, list=8%, signal=33% | GPC6/SDC2/HSPG2/SDC3/GPC1 |
| REACTOME_INTERLEUKIN_15_SIGNALING | 14 | 0.781 | 2.180 | 0.004 | | 0.033 | 0.020 | 9991 | tags=79%, list=19%, signal=64% | IL2RB/STAT5A/STAT5B/GAB2/JAK3/IL15RA/IL15/SHC1/IL2RG/JAK1/STAT3 |
| REACTOME_LGI_ADAM_INTERACTIONS | 14 | 0.787 | 2.195 | 0.004 | | 0.033 | 0.020 | 5102 | tags=57%, list=10%, signal=52% | CACNG4/LGI2/LGI4/DLG4/LGI1/ADAM22/ADAM23/STX1B |
| REACTOME_NEGATIVE_REGULATION_OF_TCF_DEPENDENT_SIGNALING_BY_WNT_LIGAND_ANTAGONISTS | 14 | 0.711 | 1.984 | 0.004 | | 0.033 | 0.020 | 3600 | tags=43%, list=7%, signal=40% | SFRP2/SFRP1/WNT9A/DKK2/SOST/WNT5A |
| REACTOME_PLATELET_ADHESION_TO_EXPOSED_COLLAGEN | 14 | 0.846 | 2.361 | 0.004 | | 0.033 | 0.020 | 4845 | tags=64%, list=9%, signal=58% | COL1A1/COL1A2/ITGA1/FCER1G/VWF/ITGB1/ITGA10/GP5/FYN |
| REACTOME_YAP1_AND_WWTR1_TAZ_STIMULATED_GENE_EXPRESSION | 14 | 0.758 | 2.116 | 0.004 | | 0.033 | 0.020 | 5202 | tags=50%, list=10%, signal=45% | NKX2-5/TBX5/WWTR1/TEAD1/RUNX2/TEAD3/TEAD2 |
| BIOCARTA_BARRESTIN_SRC_PATHWAY | 16 | 0.775 | 2.263 | 0.004 | | 0.033 | 0.020 | 4473 | tags=31%, list=9%, signal=29% | KCNA1/HCK/FGR/KCNA2/DNM1 |
