# Supplementary material for: HTRA3 Is a Prognostic Biomarker and Associated With Immune Infiltrates in Gastric Cancer
Source: Front Oncol. 2020 Dec 23;10:603480. doi: 10.3389/fonc.2020.603480 (PMC7786138; doi:10.3389/fonc.2020.603480)
Supplement: Supplemental Table 1 — Clinical characteristics of gastric cancer patients based on TCGA. [file DataSheet_1.zip › Supplemental Table 5ú║50 items of protein to protein interactions of HTRA3..docx]

Supplemental table 3：50 items of protein to protein interactions of HTRA3

| node1 | node2 | node1_external_id | node2_external_id | neighborhood_on_chromosome | gene_fusion | phylogenetic_cooccurrence | coexpression | experimentally_determined_interaction | database_annotated | automated_textmining | combined_score |
| --- | --- | --- | --- | --- | --- | --- | --- | --- | --- | --- | --- |
| KRT5 | KRT14 | ENSP00000252242 | ENSP00000167586 | 0 | 0 | 0.000000 | 0.742 | 0.991 | 0.9 | 0.208050 | 0.999 |
| COL1A2 | COL1A1 | ENSP00000297268 | ENSP00000225964 | 0 | 0 | 0.018606 | 0.991 | 0.454 | 0.9 | 0.039816 | 0.999 |
| FGA | FGB | ENSP00000306361 | ENSP00000306099 | 0 | 0 | 0.000000 | 0.992 | 0.961 | 0.9 | 0.244480 | 0.999 |
| FGG | FGB | ENSP00000336829 | ENSP00000306099 | 0 | 0 | 0.000000 | 0.992 | 0.962 | 0.9 | 0.184786 | 0.999 |
| FGG | FGA | ENSP00000336829 | ENSP00000306361 | 0 | 0 | 0.000000 | 0.993 | 0.966 | 0.9 | 0.221840 | 0.999 |
| TACR2 | TAC1 | ENSP00000362403 | ENSP00000321106 | 0 | 0 | 0.000000 | 0.061 | 0.667 | 0.9 | 0.983000 | 0.999 |
| ORM2 | ORM1 | ENSP00000394936 | ENSP00000259396 | 0 | 0 | 0.000000 | 0.954 | 0.837 | 0.9 | 0.015419 | 0.999 |
| MYLK | MYL9 | ENSP00000353452 | ENSP00000279022 | 0 | 0 | 0.000000 | 0.289 | 0.553 | 0.9 | 0.943000 | 0.997 |
| APOA2 | APOC3 | ENSP00000356969 | ENSP00000227667 | 0 | 0 | 0.000000 | 0.796 | 0.000 | 0.9 | 0.860000 | 0.996 |
| COL3A1 | COL1A2 | ENSP00000304408 | ENSP00000297268 | 0 | 0 | 0.030800 | 0.946 | 0.109 | 0.9 | 0.060130 | 0.995 |
| COL3A1 | COL1A1 | ENSP00000304408 | ENSP00000225964 | 0 | 0 | 0.021511 | 0.944 | 0.000 | 0.9 | 0.044149 | 0.994 |
| ACTA2 | MYH11 | ENSP00000402373 | ENSP00000379616 | 0 | 0 | 0.000000 | 0.339 | 0.568 | 0.9 | 0.784000 | 0.993 |
| HAND2 | NKX2-5 | ENSP00000352565 | ENSP00000327758 | 0 | 0 | 0.000000 | 0.094 | 0.263 | 0.9 | 0.895000 | 0.992 |
| KCNMA1 | KCNMB1 | ENSP00000286628 | ENSP00000274629 | 0 | 0 | 0.000000 | 0.000 | 0.472 | 0.9 | 0.811000 | 0.989 |
| AFP | APOA2 | ENSP00000379138 | ENSP00000356969 | 0 | 0 | 0.000000 | 0.875 | 0.000 | 0.9 | 0.237000 | 0.989 |
| ADIPOQ | FABP4 | ENSP00000389814 | ENSP00000256104 | 0 | 0 | 0.000000 | 0.186 | 0.000 | 0.9 | 0.879000 | 0.989 |
| ATP4B | ATP4A | ENSP00000334216 | ENSP00000262623 | 0 | 0 | 0.000000 | 0.061 | 0.417 | 0.9 | 0.809000 | 0.988 |
| VIP | CALCA | ENSP00000356213 | ENSP00000331746 | 0 | 0 | 0.000000 | 0.061 | 0.000 | 0.9 | 0.879000 | 0.987 |
| COL5A1 | COL1A1 | ENSP00000360882 | ENSP00000225964 | 0 | 0 | 0.063000 | 0.772 | 0.361 | 0.9 | 0.116100 | 0.986 |
| ACTG2 | MYH11 | ENSP00000386857 | ENSP00000379616 | 0 | 0 | 0.000000 | 0.566 | 0.325 | 0.9 | 0.596000 | 0.986 |
| TIMP3 | MMP2 | ENSP00000266085 | ENSP00000219070 | 0 | 0 | 0.000000 | 0.288 | 0.457 | 0.8 | 0.838000 | 0.985 |
| TAC1 | GRP | ENSP00000321106 | ENSP00000256857 | 0 | 0 | 0.000000 | 0.062 | 0.000 | 0.9 | 0.862000 | 0.985 |
| IVL | SPRR3 | ENSP00000357753 | ENSP00000330391 | 0 | 0 | 0.000000 | 0.283 | 0.379 | 0.9 | 0.716000 | 0.985 |
| COL5A1 | COL1A2 | ENSP00000360882 | ENSP00000297268 | 0 | 0 | 0.068160 | 0.726 | 0.406 | 0.9 | 0.114400 | 0.985 |
| SPRR2A | SPRR2E | ENSP00000376423 | ENSP00000357740 | 0 | 0 | 0.000000 | 0.544 | 0.000 | 0.9 | 0.672000 | 0.983 |
| SPRR2D | SPRR2E | ENSP00000357746 | ENSP00000357740 | 0 | 0 | 0.000000 | 0.521 | 0.000 | 0.9 | 0.669000 | 0.982 |
| SPRR2A | SPRR2D | ENSP00000376423 | ENSP00000357746 | 0 | 0 | 0.000000 | 0.526 | 0.000 | 0.9 | 0.669000 | 0.982 |
| MYH11 | MYL9 | ENSP00000379616 | ENSP00000279022 | 0 | 0 | 0.000000 | 0.202 | 0.316 | 0.9 | 0.720000 | 0.982 |
| LCE3E | SPRR2B | ENSP00000357778 | ENSP00000357744 | 0 | 0 | 0.000000 | 0.520 | 0.000 | 0.9 | 0.652000 | 0.981 |
| ATP4B | ATP12A | ENSP00000334216 | ENSP00000218548 | 0 | 0 | 0.000000 | 0.061 | 0.348 | 0.9 | 0.725000 | 0.980 |
| LCE3D | SPRR2G | ENSP00000357776 | ENSP00000357737 | 0 | 0 | 0.000000 | 0.457 | 0.000 | 0.9 | 0.678000 | 0.980 |
| IVL | TGM1 | ENSP00000357753 | ENSP00000206765 | 0 | 0 | 0.000000 | 0.098 | 0.000 | 0.9 | 0.793000 | 0.979 |
| ADCYAP1R1 | VIP | ENSP00000483721 | ENSP00000356213 | 0 | 0 | 0.000000 | 0.107 | 0.176 | 0.9 | 0.750000 | 0.979 |
| MYH11 | MYLK | ENSP00000379616 | ENSP00000353452 | 0 | 0 | 0.000000 | 0.256 | 0.193 | 0.9 | 0.686000 | 0.978 |
| LCE3D | SPRR2B | ENSP00000357776 | ENSP00000357744 | 0 | 0 | 0.000000 | 0.371 | 0.000 | 0.9 | 0.678000 | 0.977 |
| LCE3E | SPRR2G | ENSP00000357778 | ENSP00000357737 | 0 | 0 | 0.000000 | 0.521 | 0.000 | 0.9 | 0.574000 | 0.977 |
| ACTA2 | MYL9 | ENSP00000402373 | ENSP00000279022 | 0 | 0 | 0.000000 | 0.356 | 0.291 | 0.9 | 0.519000 | 0.975 |
| MUC15 | MUC21 | ENSP00000416753 | ENSP00000365473 | 0 | 0 | 0.000000 | 0.000 | 0.000 | 0.9 | 0.767000 | 0.975 |
| SPRR2E | SPRR2G | ENSP00000357740 | ENSP00000357737 | 0 | 0 | 0.000000 | 0.477 | 0.000 | 0.9 | 0.543000 | 0.974 |
| SPRR2B | SPRR2E | ENSP00000357744 | ENSP00000357740 | 0 | 0 | 0.000000 | 0.483 | 0.000 | 0.9 | 0.539000 | 0.974 |
| SPRR2F | SPRR2D | ENSP00000418193 | ENSP00000357746 | 0 | 0 | 0.000000 | 0.214 | 0.000 | 0.9 | 0.706000 | 0.974 |
| PLIN1 | FABP4 | ENSP00000300055 | ENSP00000256104 | 0 | 0 | 0.000000 | 0.159 | 0.000 | 0.9 | 0.715000 | 0.973 |
| ACTA2 | MYLK | ENSP00000402373 | ENSP00000353452 | 0 | 0 | 0.000000 | 0.260 | 0.186 | 0.9 | 0.608000 | 0.973 |
| FGG | ORM1 | ENSP00000336829 | ENSP00000259396 | 0 | 0 | 0.000000 | 0.606 | 0.000 | 0.9 | 0.354000 | 0.972 |
| APOA2 | FGA | ENSP00000356969 | ENSP00000306361 | 0 | 0 | 0.000000 | 0.628 | 0.000 | 0.9 | 0.329000 | 0.972 |
| AFP | FGG | ENSP00000379138 | ENSP00000336829 | 0 | 0 | 0.000000 | 0.684 | 0.000 | 0.9 | 0.203000 | 0.972 |
| SPRR2D | SPRR2B | ENSP00000357746 | ENSP00000357744 | 0 | 0 | 0.000000 | 0.292 | 0.000 | 0.9 | 0.630000 | 0.971 |
| COL1A1 | MMP2 | ENSP00000225964 | ENSP00000219070 | 0 | 0 | 0.000000 | 0.890 | 0.379 | 0.0 | 0.600000 | 0.970 |
| FGA | ORM1 | ENSP00000306361 | ENSP00000259396 | 0 | 0 | 0.000000 | 0.488 | 0.000 | 0.9 | 0.472000 | 0.970 |
| APOA2 | FGG | ENSP00000356969 | ENSP00000336829 | 0 | 0 | 0.000000 | 0.639 | 0.000 | 0.9 | 0.239000 | 0.970 |
