# Supplementary material for: HTRA3 Is a Prognostic Biomarker and Associated With Immune Infiltrates in Gastric Cancer
Source: Front Oncol. 2020 Dec 23;10:603480. doi: 10.3389/fonc.2020.603480 (PMC7786138; doi:10.3389/fonc.2020.603480)
Supplement: Supplemental Table 1 — Clinical characteristics of gastric cancer patients based on TCGA. [file DataSheet_1.zip › Supplemental Table 6ú║Univariate regression and multivariate survival method (Disease Specific Survival) of prognostic covariates in patients with gastric cancer..docx]

| Characteristics | Total(N) | HR(95% CI) Univariate analysis | P value Univariate analysis | HR(95% CI) Multivariate analysis | P value Multivariate analysis |
| --- | --- | --- | --- | --- | --- |
| T stage (T3&T4 vs. T1&T2) | 345 | 2.089(1.192-3.660) | 0.010 | 1.160(0.553-2.432) | 0.694 |
| N stage (N1&N2&N3 vs. N0) | 334 | 1.807(1.075-3.036) | 0.025 | 1.575(0.566-4.381) | 0.384 |
| M stage (M1 vs. M0) | 333 | 2.438(1.221-4.870) | 0.012 | 0.677(0.276-1.661) | 0.394 |
| Pathologic stage (Stage III&Stage IV vs. Stage I&Stage II) | 331 | 2.146(1.352-3.404) | 0.001 | 0.931(0.381-2.277) | 0.875 |
| Histologic grade (G3 vs. G1&G2) | 340 | 1.338(0.862-2.078) | 0.194 |  |  |
| Histological type (Diffuse Type vs. Tubular Type) | 129 | 1.115(0.597-2.082) | 0.734 |  |  |
| Primary therapy outcome (CR vs. PD&SD&PR) | 310 | 0.115(0.072-0.184) | <0.001 | 0.509(0.276-0.938) | 0.030 |
| Residual tumor (R1&R2 vs. R0) | 314 | 5.142(3.014-8.771) | <0.001 | 2.188(1.105-4.333) | 0.025 |
| Age (>65 vs. <=65) | 346 | 1.211(0.797-1.840) | 0.371 |  |  |
| Race (Asian&Black or African American vs. White) | 305 | 1.097(0.656-1.836) | 0.724 |  |  |
| Gender (Male vs. Female) | 349 | 1.573(0.985-2.514) | 0.058 | 1.181(0.669-2.085) | 0.566 |
| Anatomic neoplasm subdivision (Fundus/Body vs. Antrum/Distal) | 253 | 0.850(0.512-1.412) | 0.531 |  |  |
| Reflux history (Yes vs. No) | 208 | 0.598(0.272-1.313) | 0.200 |  |  |
| Antireflux treatment (Yes vs. No) | 167 | 0.758(0.380-1.511) | 0.431 |  |  |
| Barretts esophagus (Yes vs. No) | 201 | 0.974(0.304-3.118) | 0.964 |  |  |
| TP53 status (Mut vs. WT) | 346 | 1.007(0.662-1.532) | 0.974 |  |  |
| PIK3CA status (Mut vs. WT) | 346 | 0.815(0.452-1.470) | 0.497 |  |  |
| Tumor status (With tumor vs. Tumor free) | 332 | 96.460(23.678-392.970) | <0.001 | 106.239(14.035-804.160) | <0.001 |
| HTRA3 (High vs. Low) | 349 | 1.650(1.079-2.522) | 0.021 | 1.405(0.824-2.395) | 0.211 |
