# Supplementary material for: HTRA3 Is a Prognostic Biomarker and Associated With Immune Infiltrates in Gastric Cancer
Source: Front Oncol. 2020 Dec 23;10:603480. doi: 10.3389/fonc.2020.603480 (PMC7786138; doi:10.3389/fonc.2020.603480)
Supplement: Supplemental Table 1 — Clinical characteristics of gastric cancer patients based on TCGA. [file DataSheet_1.zip › Supplemental Table 7ú║Univariate regression and multivariate survival method (Progression Free Interval) of prognostic covariates in patients with gastric cancer..docx]

| Characteristics | Total(N) | HR(95% CI) Univariate analysis | P value Univariate analysis | HR(95% CI) Multivariate analysis | P value Multivariate analysis |
| --- | --- | --- | --- | --- | --- |
| T stage (T3&T4 vs. T1&T2) | 364 | 1.705(1.095-2.654) | 0.018 | 0.629(0.283-1.399) | 0.256 |
| N stage (N1&N2&N3 vs. N0) | 354 | 1.640(1.075-2.501) | 0.022 | 2.417(0.833-7.012) | 0.104 |
| M stage (M1 vs. M0) | 353 | 2.224(1.194-4.144) | 0.012 | 0.832(0.283-2.444) | 0.738 |
| Pathologic stage (Stage III&Stage IV vs. Stage I&Stage II) | 349 | 1.676(1.154-2.435) | 0.007 | 0.508(0.178-1.452) | 0.206 |
| Histologic grade (G3 vs. G1&G2) | 363 | 1.540(1.057-2.245) | 0.025 | 1.323(0.748-2.340) | 0.336 |
| Histological type (Diffuse Type vs. Tubular Type) | 132 | 1.241(0.719-2.144) | 0.438 |  |  |
| Primary therapy outcome (CR vs. PD&SD&PR) | 315 | 0.124(0.085-0.183) | <0.001 | 0.456(0.241-0.862) | 0.016 |
| Residual tumor (R1&R2 vs. R0) | 326 | 3.469(2.127-5.656) | <0.001 | 1.879(0.887-3.981) | 0.099 |
| Age (>65 vs. <=65) | 369 | 0.858(0.603-1.221) | 0.395 |  |  |
| Race (Asian&Black or African American vs. White) | 322 | 1.061(0.688-1.637) | 0.787 |  |  |
| Gender (Male vs. Female) | 372 | 1.638(1.099-2.440) | 0.015 | 0.820(0.417-1.615) | 0.566 |
| Anatomic neoplasm subdivision (Fundus/Body vs. Antrum/Distal) | 267 | 0.728(0.470-1.128) | 0.156 |  |  |
| Reflux history (Yes vs. No) | 214 | 0.482(0.232-1.000) | 0.050 | 0.986(0.274-3.554) | 0.983 |
| Antireflux treatment (Yes vs. No) | 179 | 0.584(0.298-1.146) | 0.118 |  |  |
| Barretts esophagus (Yes vs. No) | 208 | 0.953(0.348-2.612) | 0.926 |  |  |
| TP53 status (Mut vs. WT) | 369 | 1.061(0.744-1.514) | 0.743 |  |  |
| PIK3CA status (Mut vs. WT) | 369 | 0.895(0.549-1.460) | 0.657 |  |  |
| Tumor status (With tumor vs. Tumor free) | 335 | 46.359(21.507-99.930) | <0.001 | 65.436(14.652-292.235) | <0.001 |
| HTRA3 (High vs. Low) | 372 | 1.456(1.021-2.078) | 0.038 | 1.116(0.656-1.898) | 0.686 |

DFI
