# Supplementary material for: HTRA3 Is a Prognostic Biomarker and Associated With Immune Infiltrates in Gastric Cancer
Source: Front Oncol. 2020 Dec 23;10:603480. doi: 10.3389/fonc.2020.603480 (PMC7786138; doi:10.3389/fonc.2020.603480)
Supplement: Supplemental Table 1 — Clinical characteristics of gastric cancer patients based on TCGA. [file DataSheet_1.zip › Supplemental Table 8ú║The prognostic value of HTRA3 (Disease Specific Survival) in various gastric cancer subgroups..docx]

| Characteristics | N (%) | HR(95% CI) | P value |
| --- | --- | --- | --- |
| T stage |  |  |  |
| T1&T2 | 90 (26) | 1.087(0.386-3.058) | 0.875 |
| T3 | 158 (46) | 1.486(0.824-2.680) | 0.188 |
| T4 | 97 (28) | 1.780(0.793-3.996) | 0.162 |
| N stage |  |  |  |
| N0 | 104 (31) | 2.225(0.792-6.250) | 0.129 |
| N1 | 88 (26) | 1.722(0.691-4.293) | 0.244 |
| N2&N3 | 142 (43) | 1.644(0.929-2.909) | 0.088 |
| M stage |  |  |  |
| M0 | 311 (93) | 1.665(1.055-2.627) | 0.029 |
| M1 | 22 (7) | 1.079(0.283-4.114) | 0.911 |
| Pathologic stage |  |  |  |
| Stage I | 48 (15) | 0.935(0.155-5.650) | 0.942 |
| Stage II | 106 (32) | 2.016(0.781-5.204) | 0.148 |
| Stage III&Stage IV | 177 (53) | 1.595(0.946-2.687) | 0.080 |

DSS
