# Supplementary material for: HTRA3 Is a Prognostic Biomarker and Associated With Immune Infiltrates in Gastric Cancer
Source: Front Oncol. 2020 Dec 23;10:603480. doi: 10.3389/fonc.2020.603480 (PMC7786138; doi:10.3389/fonc.2020.603480)
Supplement: Supplemental Table 1 — Clinical characteristics of gastric cancer patients based on TCGA. [file DataSheet_1.zip › Supplemental Table 9ú║The prognostic value of HTRA3 (Progression Free Interval) in various gastric cancer subgroups..docx]

DFI

| Characteristics | N (%) | HR(95% CI) | P value |
| --- | --- | --- | --- |
| T stage |  |  |  |
| T1&T2 | 97 (27) | 1.366(0.619-3.012) | 0.440 |
| T3 | 168 (46) | 1.363(0.837-2.219) | 0.214 |
| T4 | 99 (27) | 1.238(0.598-2.563) | 0.565 |
| N stage |  |  |  |
| N0 | 108 (31) | 1.714(0.791-3.714) | 0.172 |
| N1 | 97 (27) | 1.419(0.684-2.946) | 0.348 |
| N2&N3 | 149 (42) | 1.618(0.978-2.675) | 0.061 |
| M stage |  |  |  |
| M0 | 328 (93) | 1.361(0.933-1.986) | 0.110 |
| M1 | 25 (7) | 2.058(0.608-6.970) | 0.246 |
| Pathologic stage |  |  |  |
| Stage I | 51 (15) | 0.982(0.244-3.958) | 0.980 |
| Stage II | 110 (32) | 1.602(0.796-3.224) | 0.187 |
| Stage III&Stage IV | 188 (54) | 1.496(0.940-2.379) | 0.089 |
